# Human activities modulate reciprocal effects of a subterranean ecological engineer rodent, *Tachyoryctes macrocephalus*, on Afroalpine vegetation cover

**DOI:** 10.1002/ece3.10337

**Published:** 2023-07-17

**Authors:** Addisu Asefa, Victoria Reuber, Georg Miehe, Luise Wraase, Tilaye Wube, Dana G. Schabo, Nina Farwig

**Affiliations:** ^1^ Department of Biology, Conservation Ecology Philipps‐Universität Marburg Marburg Germany; ^2^ Department of Geography, Vegetation Geography Philipps‐Universität Marburg Marburg Germany; ^3^ Department of Geography, Environmental Informatics Philipps‐Universität Marburg Marburg Germany; ^4^ Department of Zoology, College of Natural and Computational Sciences Addis Ababa University Addis Ababa Ethiopia

**Keywords:** bioturbation, grazing intensity, human settlement, rodent burrow, synanthropic association

## Abstract

Human activities, directly and indirectly, impact ecological engineering activities of subterranean rodents. As engineering activities of burrowing rodents are affected by, and reciprocally affect vegetation cover via feeding, burrowing and mound building, human influence such as settlements and livestock grazing, could have cascading effects on biodiversity and ecosystem processes such as bioturbation. However, there is limited understanding of the relationship between human activities and burrowing rodents. The aim of this study was therefore to understand how human activities influence the ecological engineering activity of the giant root‐rat (*Tachyoryctes macrocephalus*), a subterranean rodent species endemic to the Afroalpine ecosystem of the Bale Mountains of Ethiopia. We collected data on human impact, burrowing activity and vegetation during February and March of 2021. Using path analysis, we tested (1) direct effects of human settlement on the patterns of livestock grazing intensity, (2) direct and indirect impacts of humans and livestock grazing intensity on the root‐rat burrow density and (3) whether human settlement and livestock grazing influence the effects of giant root‐rat burrow density on vegetation and vice versa. We found lower levels of livestock grazing intensity further from human settlement than in its proximity. We also found a significantly increased giant root‐rat burrow density with increasing livestock grazing intensity. Seasonal settlement and livestock grazing intensity had an indirect negative and positive effect on giant root‐rat burrow density, respectively, both via vegetation cover. Analysing the reciprocal effects of giant root‐rat on vegetation, we found a significantly decreased vegetation cover with increasing density of giant root‐rat burrows, and indirectly with increasing livestock grazing intensity via giant root‐rat burrow density. Our results demonstrate that giant root‐rats play a synanthropic engineering role that affects vegetation structure and ecosystem processes.

## Graphical abstract

In our study, we tested how human activities in terms of livestock grazing and settlements affect the reciprocal effects between vegetation and giant root‐rats. Our results show that increasing livestock grazing intensity causes increases in giant root‐rat burrow density indirectly through decreased vegetation cover. Reciprocally, the root‐rat's engineering activities resulted in decreased vegetation cover.

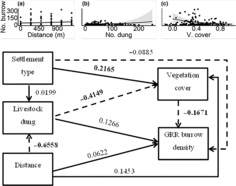

## 1 INTRODUCTION

Human‐induced land use change is the main cause of biodiversity loss and disruption of ecosystem processes globally (Díaz et al., 2019). One of the most extensive land‐use types worldwide, and thus one of the most detrimental for biodiversity, is livestock production (Eldridge et al., 2016; FAO, 2018; Filazzola et al., 2020) although certain types of livestock production can be less detrimental than intensive crop agriculture or urbanization (Olivier et al., 2020). Currently, livestock grazing occupies 26% of global terrestrial land cover (FAO, 2018). The increasing global livestock population (Bar‐On et al., 2018), but declining extent and productivity of rangelands, has resulted in changes in traditional grazing practices in Africa (FAO, 2018), such as rotational grazing and seasonal movements, to partly sedentary grazing systems (Bagchi et al., 2006; Reitalu et al., 2010). In contrast, seasonal grazing systems that are evenly distributed across rangelands, livestock grazing intensity in sedentary grazing systems and its influences on biodiversity are largely concentrated to nearby human settlements, declining as a function of increasing distance from settlement areas (Bagchi et al., 2006; Dunne et al., 2011; Reitalu et al., 2010).

Livestock have strong direct and indirect effects on biodiversity and ecosystem processes, mainly through grazing, trampling, defecation and urination (Eldridge et al., 2016; Maestre et al., 2022; Narantsetseg et al., 2018). For example, grazing by livestock directly creates bare soil, and indirectly causes soil loss via wind and rain erosion, and by facilitating the rapid run‐off of rainfall (FAO, 2018). Moreover, grazing by livestock removes plant biomass, thereby directly reducing plant cover and eliminating grazing intolerant species (Tessema et al., 2011), and indirectly by creating open spaces for gap‐colonizing plant species and promoting the dominance of unpalatable and grazing tolerant species (Eldridge et al., 2016; Niu et al., 2019; Pavlů et al., 2019; Tessema et al., 2011). Livestock trampling causes soil compaction and disruption of surface layers, which indirectly reduce infiltration and increase runoff and soil loss (Dunne et al., 2011; FAO, 2018). Further, through trampling, livestock also directly reduces vegetation cover and height, regeneration and recovery from grazing impacts by damaging seedlings and vegetative organs (Eldridge et al., 2016). Livestock trampling also indirectly affects vegetation via changes in soil properties (Tessema et al., 2011). Finally, livestock dung deposition and urination affect nutrient cycling and can cause nutrient overloading, which affects vegetation structure and diversity by facilitating encroachment of exploitive native and/or non‐native plant species that may lead to biotic homogenization (Bokdam, 2001; Dunne et al., 2011; Pavlů et al., 2019). Therefore, livestock production may result in changes in vegetation structure and composition, and reduced or increased plant species diversity, depending on the intensity of grazing, trampling and degree of resistance of the regional species pool to different grazing intensities (Eldridge et al., 2016; FAO, 2018).

Apart from the effects on soil and vegetation, these direct and indirect effects of human activities related to settlement establishment and livestock grazing cascade to wild animals, affecting their distribution, abundance and behaviour (Wang et al., 2020). Subterranean small rodents that are adapted to living in savannah and alpine grassland ecosystems are particularly known to be susceptible to human activities (Vial et al., 2010; Wang et al., 2019, 2020). For example, habitat loss and degradation caused by human settlement—via space use for house building—and livestock husbandry—via grazing and trampling—lead to deterioration of habitat quality for rodents (Bakker et al., 2009). Moreover, livestock grazing can lead to competition for food with rodents (Niu et al., 2019; Zhang & Liu, 2003), and livestock trampling can destroy burrow systems (Šklíba et al., 2017). Despite these generally presumed negative effects, livestock grazing and trampling activities can also have positive effects on subterranean rodents inhabiting grasslands through facilitation of habitat by reducing vegetation height and cover (Asefa et al., 2022; Bakker et al., 2009). Thus, while grazing can affect both vegetation and subterranean engineering animals, whether the impacts would be positive or negative are dependent on the local context in relation to livestock density.

Effects on subterranean rodents can also cascade through the system, as many burrowing rodents are ecosystem engineers, transforming ecosystems through their feeding and burrowing activities (Beyene, 1986; Davidson et al., 2012; Jones et al., 1997). They can directly reduce vegetation cover and diversity through consumption of plants and burrowing and mound building activities that bury vegetation under the excavated soil (Šklíba et al., 2017; Wang et al., 2019). Ejection of soil, decomposed cached foods and defecations from their underground tunnels onto the ground surface lead to redistribution of soil moisture and air, alteration of nutrient availability, and increased microhabitat heterogeneity (Haussmann, 2017; Reichman & Seabloom, 2002; Zhang & Liu, 2003). Thus, by providing new spaces and nutrient‐rich microhabitat, subterranean rodents facilitate colonization by new plant species, potentially leading to increased diversity (Hagenah & Bennett, 2013; Jones et al., 1997; Reichman & Seabloom, 2002; Šklíba et al., 2017). As most such rodents prefer low vegetation cover and they also lower vegetation cover, subterranean rodents and vegetation reciprocally affect each other (Asefa et al., 2022). At the same time, while subterranean rodents shape vegetation patterns, their activity strongly depends on vegetation (Huntly & Reichman, 1994; Zhang & Liu, 2003). For instance, the activities of rodents in dry regions are shown to increase with increasing vegetation cover (Zhang & Liu, 2003), and plant productivity is also shown to positively affect the abundance of rodents (Šklíba et al., 2017). On the contrary, the activities of rodents in alpine regions are shown to decrease with increasing vegetation cover (Asefa et al., 2022; Wang et al., 2020). Thus, given the direct and indirect effects of human activities both on vegetation and subterranean rodents, this natural interplay between vegetation and rodents is sensitive to human activities (Eldridge & Soliveres, 2023; Jones, 2012). Many of the burrowing rodents are critically endangered and vulnerable in many parts of the world, and as many of them are ecosystem engineers, their decline has a disproportionally large effect on other components of the habitats they live in, such as on soil properties and vegetation dynamics (Eldridge & Soliveres, 2023; Valkó et al., 2022). In addition to their impact on soil and vegetation, engineer rodents also impact other animals relying on vegetation for food, shelter and reproduction (Jones, 2012). Yet, there have been limited understandings on the nature and extent of such complex interactions between human activities, vegetation and subterranean rodents (Eldridge & Soliveres, 2023; Valkó et al., 2022); specifically, how the effects of human activities on vegetation affect rodents engineering activities and its reciprocal effects on vegetation.

In this study, we examined the influences of human activities on the reciprocal effects between vegetation and the ecosystem engineering activities of an endemic subterranean rodent, the giant root‐rat (*Tachyoryctes macrocephalus*, rüppell 1842) in the Afroalpine grassland and moorland ecosystem of the Bale Mountains in southeastern Ethiopia. Human associations with giant root‐rats in the Bale Mountains date back to 43–47 thousand years ago, where the middle Stone Age foragers used to hunt the root‐rats (Ossendorf et al., 2019) although the root‐rats are neither hunted nor considered as pest in the present times. Consequently, it has been supposed that giant root‐rats have a synanthropic association with human activities (Ossendorf et al., 2019). In recent decades, the numbers of human settlements and livestock in the mountains have grown rapidly (Johansson & Granström, 2014; Vial et al., 2010), with some scenarios even showing the grazing level to approach the ecosystem collapse threshold (BMNP, 2017; Vial et al., 2011). There are two types of settlements in the mountains: permanent settlement and seasonal settlements, which occur in the wetter months, from April to August, when livestock are moved from lower pastures where agricultural crops are being grown (BMNP, 2017; Hillman, 1986). As such, livestock grazing intensity and other possible human activities would differ between settlement types and vary along the distance from settlements. Here, we evaluated: (1) livestock grazing intensity in relation to human settlement type, differentiating between traditional seasonal versus permanent and along a distance gradient from the settlements, (2) the direct and indirect (via vegetation variables) effects of human settlement type, distance from settlement and livestock grazing intensity on giant root‐rat burrow density; and (3) the indirect influences of human settlement and livestock grazing intensity on the reciprocal effects of giant root‐rat burrowing activities on vegetation cover and plant species richness. We predicted that: (1) livestock grazing intensity would be higher at permanent human settlement areas than at seasonal settlement and decline with increasing distance from settlement, (2) permanent settlement and increasing grazing directly and indirectly lead to decreased vegetation cover which in turn leads to increased giant root‐rat burrow density, but lead to decreased plants species richness that in turn results in decreased root‐rat burrow density—clearly stating, livestock grazing decreases plant cover and diversity, and root‐rat present burrowing activities make it even worse, and (3) giant root‐rats would have negative reciprocal effects on vegetation, which in turn are influenced by human activities, on vegetation, as plant biomass damage caused by giant root‐rat burrowing and foraging activities would reduce vegetation cover and plant species richness.

## 2 MATERIALS AND METHODS

### 2.1 Study species

The giant root‐rat (*Tachyoryctes macrocephalus*, rüppell 1842, family Spalacidae; Šumbera et al., 2018) is a rodent species endemic to the Bale Mountains of Ethiopia (Lavrenchenko & Kennerley, 2016; Yalden, 1985; Yalden & Largen, 1992). The species is restricted to <1000 km^2^ area at altitudes from 3000 to 4150 m above sea level (asl; Šumbera et al., 2018), where it is the main prey of the endangered Ethiopian Wolf (*Canis simensis*) and numerous raptor species, such as golden eagle (*Aquila chrysaetos*), lesser‐spotted eagle (*A. pomarina*), tawny eagle (*A. rapax*), Verreaux's eagle (*A. verreauxi*) and augur buzzard (*Buteo rufofuscus*) (Asefa, 2007; Sillero‐Zubiri et al., 1995). The giant root‐rats are diurnal species and occur with a density of 63 animals per ha (Yalden, 1985). They construct extensive large underground burrow systems. An individual root‐rat burrow system extends up to 34 m, which branches into short tunnels that comprise nesting and food caching and defecation chambers (Beyene, 1986; Sillero‐Zubiri et al., 1995; Yaba et al., 2011). Burrow holes are used to expel soil, as well as decomposed cached foods and defecations, and to access aboveground vegetation for feeding. Unused burrow holes are plugged in by soil backfilling while all active fresh holes are plugged in during night time for thermoregulation (Beyene, 1986; Šklíba et al., 2017; Šumbera et al., 2018). It is via these burrowing and feeding activities that giant root‐rats impact ecosystem processes and vegetation structure and diversity (Asefa et al., 2022). Despite the vital ecological engineering role it plays, the species is currently classified by the IUCN as endangered mainly due to habitat loss and degradation brought about by livestock overgrazing (BMNP, 2017; Lavrenchenko & Kennerley, 2016).

### 2.2 Study area

**FIGURE 1:**
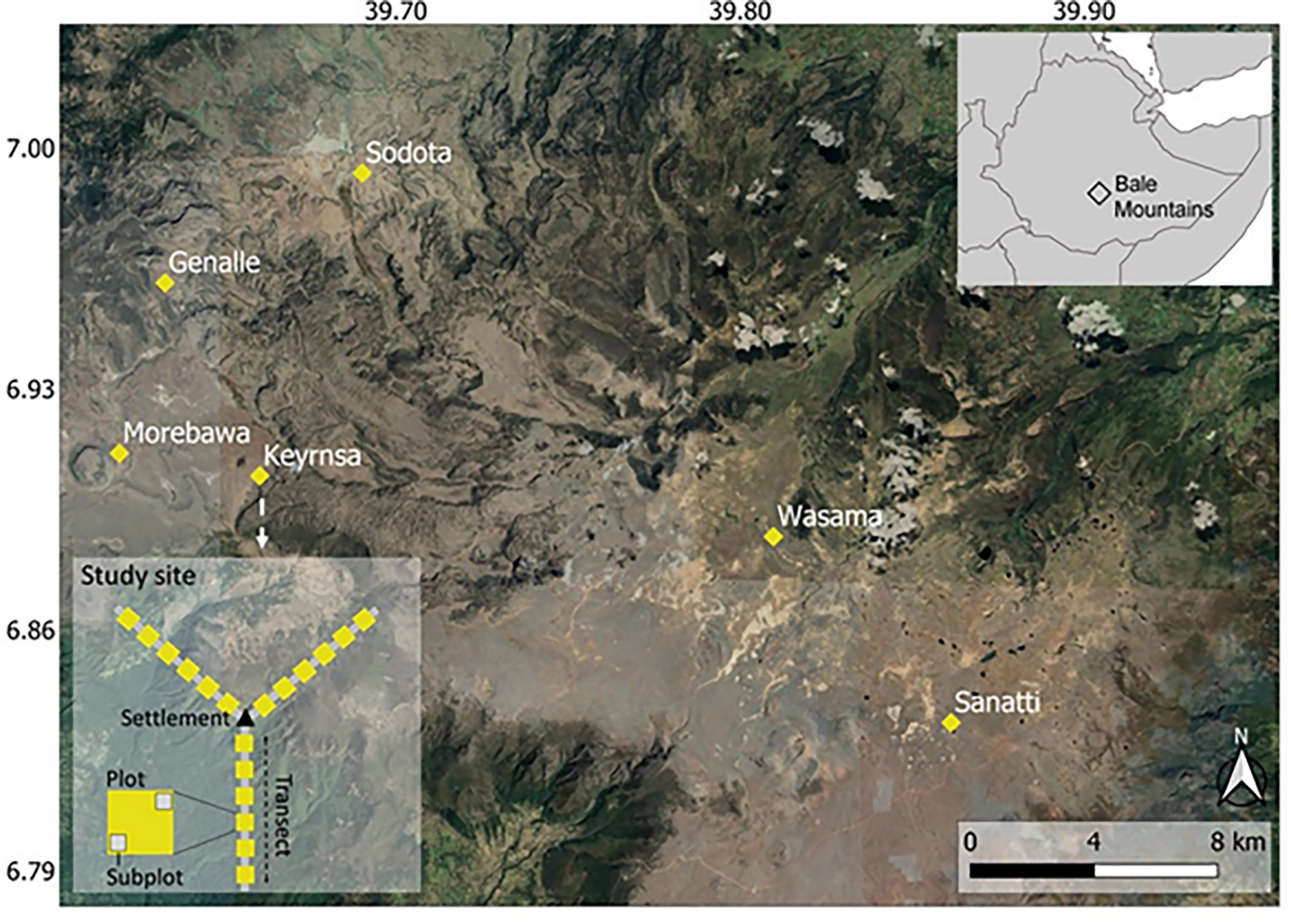
Overview map of the Bale Mountains National Park and its location in southeast Ethiopia (top‐right inset) and the six study sites, and detailed inset map (bottom‐left) showing the set‐up of one study location with three transects of 1.5 km length, six study plots along each transect and two subplots within each plot (for detailed description see Section 2.2).

This study was conducted in the Afroalpine ecosystem of the Bale Mountains National Park in southeastern Ethiopia (6.508–7.178 N, 39.508–39.928 E; Figure 1), between December 2020 and February 2021. With elevation ranging between 1500 and 4377 m asl, the Bale Mountains represent the largest area of Afroalpine vegetation over 3000 m asl in Africa (Yalden, 1983). The area experiences two rainy seasons, with lighter rains from March to June and the main rainy season from July to October, and a dry season between November and February; mean annual rainfall is approximately 1000 mm (Miehe & Miehe, 1994). The lowest and maximum recorded temperature in the Bale mountains is −15 and 26°C, respectively (Miehe & Miehe, 1994; OBARD, 2007). The soils in the Bale Mountains are entirely volcanic in origin and mainly derived from the basaltic and trachytic parent rock, are fairly fertile silty loams of reddish‐brown to black colour (Hillman, 1986; Miehe & Miehe, 1994).The Bale Mountains region is a global biodiversity hotspot area hosting a high level of endemism, including many local endemics such as the giant root‐rat (BMNP, 2017). In the region, rock‐shelters were repeatedly occupied by humans in prehistoric times and represent the world's oldest known high‐altitudinal residential site. Those prehistoric high‐altitude residents used to forage on the locally endemic giant root‐rats (Ossendorf et al., 2019). Similar to many other alpine ecosystems in Africa, more rapid ecosystem changes have been detected in the Bale Mountains over the past 40 years (BMNP, 2017; Johansson & Granström, 2014; Tallents, 2007). Reber et al. (2018) have recorded a total of 870 settlements (207 permanently inhabited, 449 seasonally inhabited, and 214 uninhabited) in the Afroalpine zone of the Bale Mountains. Socio‐economic survey conducted in 2013 show 863 households, each having an average of four people, in the study area (BMNP, unpublished data). Permanent settlers live and use the area throughout the year, while seasonal settlement occurs in the wetter months, from April to August, when livestock are moved from lower pastures where agricultural crops are being grown and thus human activities are higher (Hillman, 1986).

### 2.3 Data collection

To examine the relationships between human settlement, livestock grazing, vegetation variables and giant root‐rat burrow density, we worked across the entire distribution range of the giant root‐rat that is scattered over an area of 1000 km^2^. Six study sites were selected systematically, between 5 and 20 km apart, to cover all major sections (eastern, central and western sections; Figure 1) and vegetation types of the Bale Mountains' Afroalpine ecosystem (open grassland, grassland dotted with *Artemisia afra* shrub, *Helichrysum* dwarf‐scrub, *Alchemilla* meadow, *Lobelia rhychopetalum*, and wetlands, such as alpine lakes, rivers, swamps and seasonal wetland grasslands; Tallents, 2007). At each site, we selected two adjacent settlements (3–5 km apart) that were known to be established 30 years ago (BMNP, 2017; Hillman, 1986), one of them being permanent and one seasonal. Starting at the centre of each settlement, we established three 1.5‐km‐long transects at an angle of 80–120° (see the inset map on Figure 1). Along each transect, we established six 25 m × 25 m plots at a distance of 250 m from each other. We determined the size of plots and distance from one another to standardize and make comparable with our recent study (Asefa et al., 2022). Plots were placed within a uniform habitat type and at least 15 m away from any habitat edge. In total, there were 216 plots covering an area of 13.5 ha.

We undertook data collection during the late dry season (February and March) of 2021, but our intended wet season sampling was not possible due to logistic and security reason. At each plot, we recorded (1) two proxy variables characterizing and quantifying the intensity of grazing and other possible human activities: (1a) settlement type (seasonal vs. permanent settlement at centre), and (1b) distance from the settlement, a proxy for grazing intensity and for other possible human activities; (2) abundance of livestock dung (cattle and horses), a proxy for livestock grazing intensity; (3) giant root‐rat burrow density; and (4) vegetation variables. For giant root‐rat burrow density, we used only fresh burrows as our main interest was to reflect the species' current burrowing activity and link it to current human activities and vegetation patterns. Giant root‐rat fresh burrows are easily distinguished from old burrows in that the former are freshly open or plugged holes that are currently active. However, giant root‐rat old burrows are abandoned burrows, with holes open or plugged with weathered soil, partially or wholly covered by vegetation regrowth, and sometimes occupied by other small rodents (Asefa et al., 2022; Sillero‐Zubiri et al., 1995; Šklíba et al., 2017). We recorded vegetation data within two 10 m × 10 m subplots established at opposite corners of each plot. The size of plots and subplots was chosen to be comparable to our recent study (Asefa et al., 2022). In each of these subplots, we identified all plants to species level, except grasses that were collectively recorded as a single morpho‐species, and estimated, in 5% intervals, percentage cover of each species. Based on Miehe and Miehe's (1994) elevational distribution of plants species in the montane and alpine areas, about 10 grass species are expected to occur in the study area, which collectively have an average cover of 15%–20%. This grouping of grasses to a single morpho‐species may consistently underestimate our species richness and modulate diversity values, but we could not avoid this potential bias because many grasses were overgrazed and difficult to identify at species level during our survey. We also recorded percentage cover of overall vegetation. For analyses on the plot level, we averaged cover values of overall vegetation converted into proportion and combined species lists obtained from the two subplots and calculated the cumulative number of species (species richness).

### 2.4 Data analysis

**FIGURE 2:**
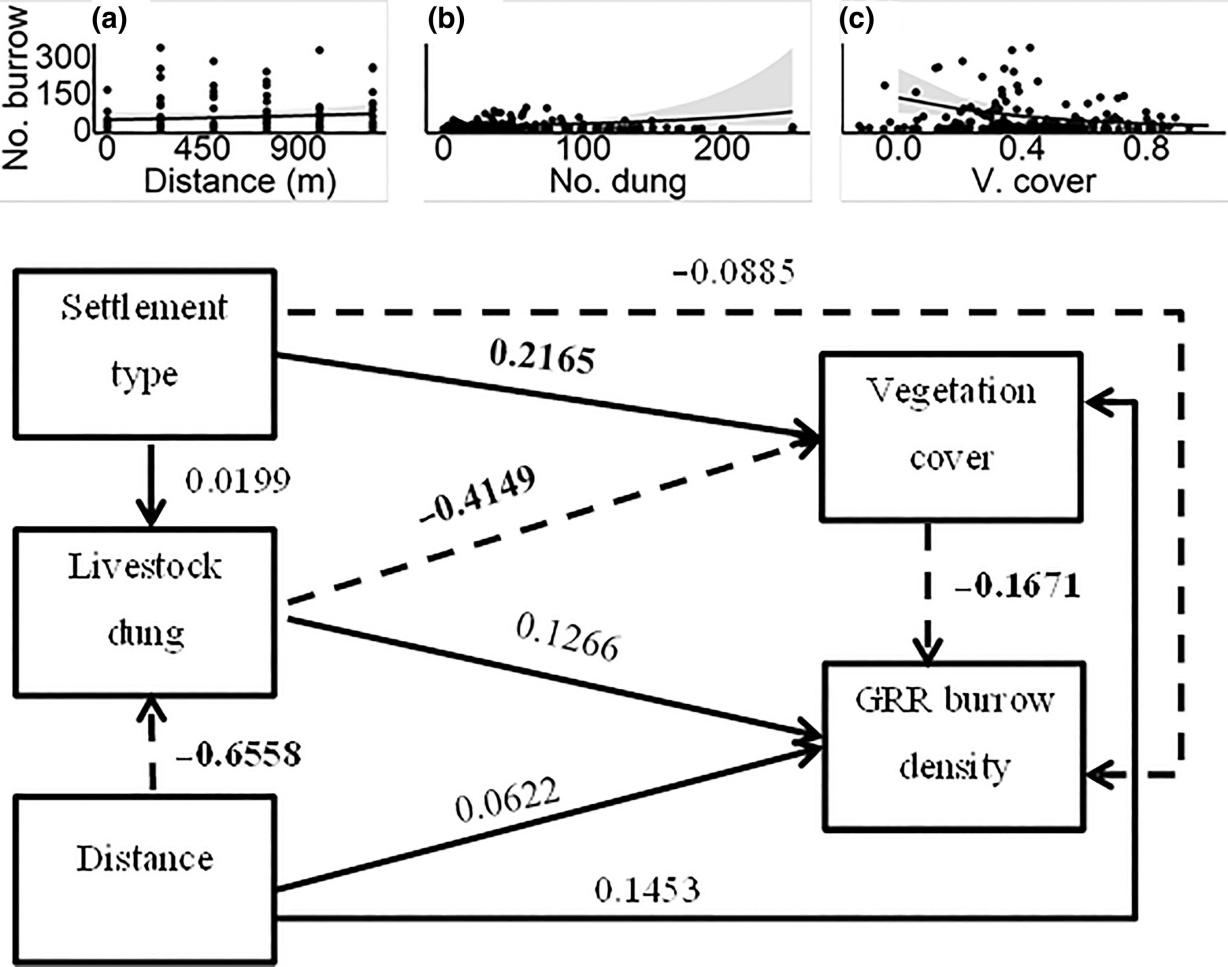
Standardized path coefficients of the direct effects of settlement type (seasonal), distance from settlement, livestock dung abundance and vegetation cover on giant root‐rat burrow density. Paths in solid line indicate positive associations, while those with broken line indicate negative associations. Path coefficients indicated in bold font denote statistically significant effect at *p* < .05. The inset figures show the relationships of distance from settlement (a), livestock dung abundance (b) and vegetation cover (c) with giant root‐rat (GRR) burrow density.

**FIGURE 3:**
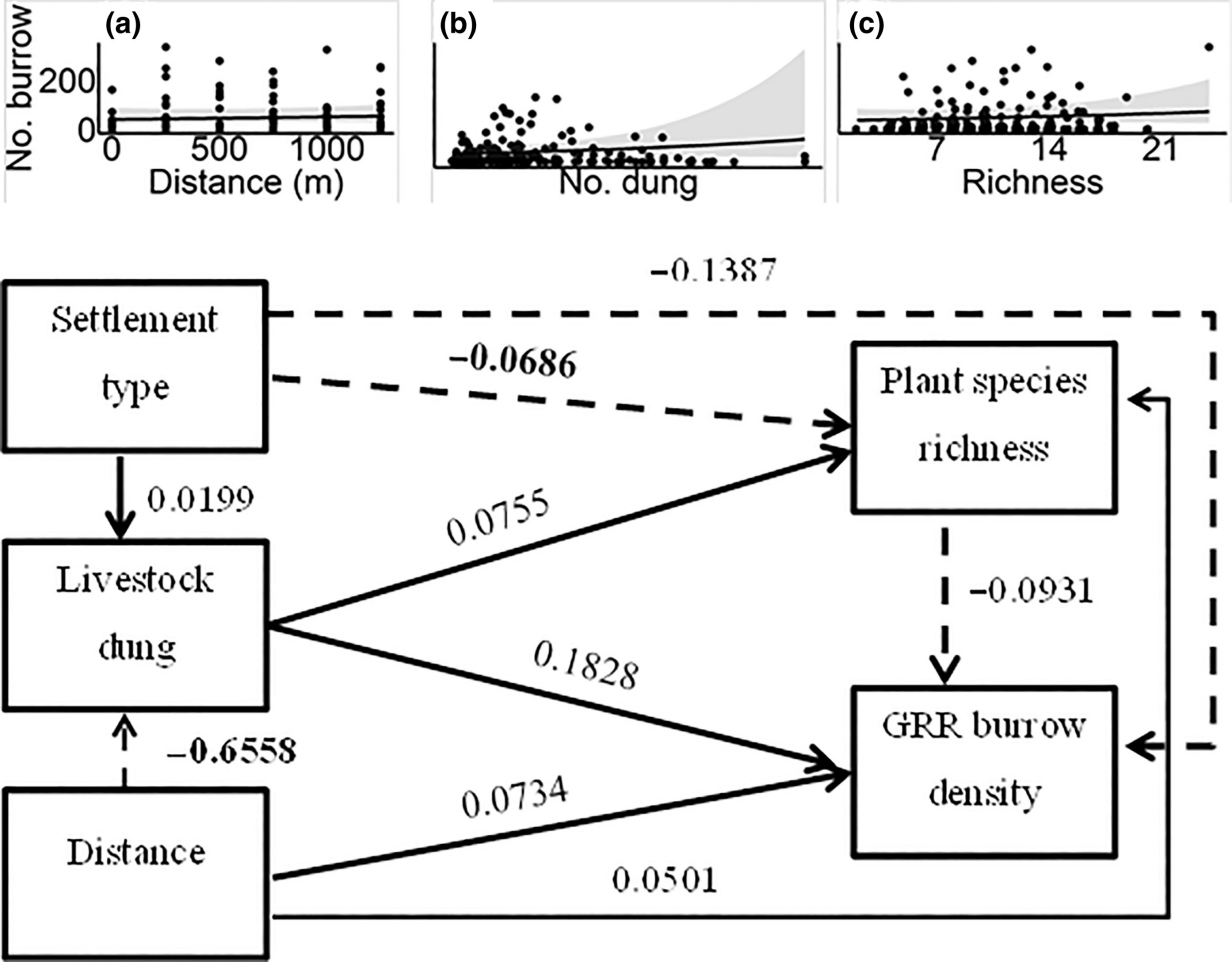
Standardized path coefficients of the direct effects of settlement type, distance from settlement, livestock dung abundance and plant species richness on giant root‐rat burrow density. Paths in solid line indicate positive associations, while those with broken line indicate negative associations. Path coefficients indicated in bold font denote statistically significant effect at *p* < .05. The inset figures show the relationships of distance from settlement (a), livestock dung abundance (b) and plant species richness (c) with giant root‐rat (GRR) burrow density.

**FIGURE 4:**
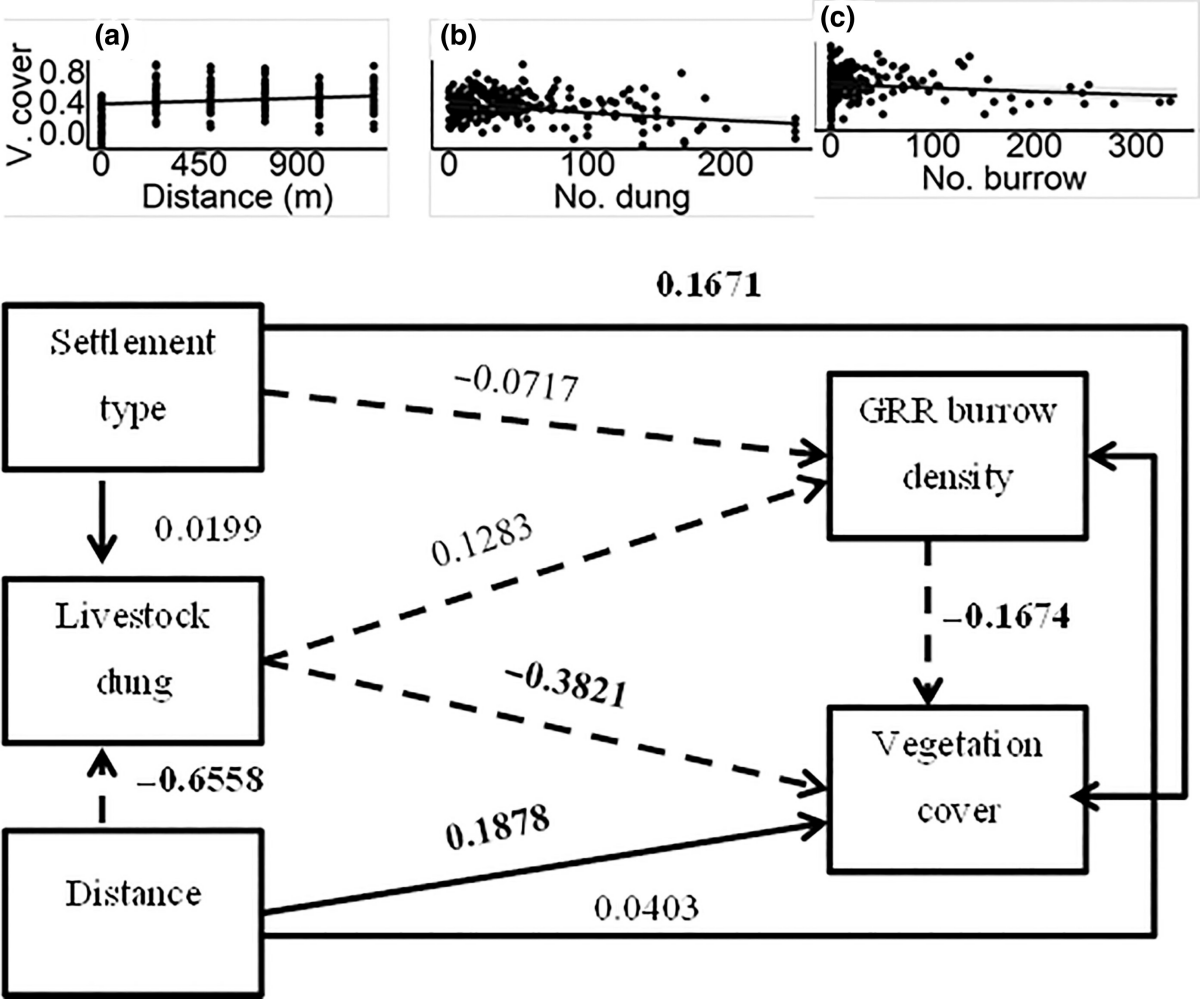
Standardized path coefficients of the direct effects of settlement type (seasonal), distance from settlement, livestock dung abundance and giant root‐rat burrow density on vegetation cover. Paths in solid line indicate positive associations, while those with broken line indicate negative associations. Path coefficients indicated in bold font denote statistically significant effect at *p* < .05. The inset figures show the relationships of distance from settlement (a), livestock dung abundance (b) and giant root‐rat (GRR) burrow density (c) with vegetation cover.

**FIGURE 5:**
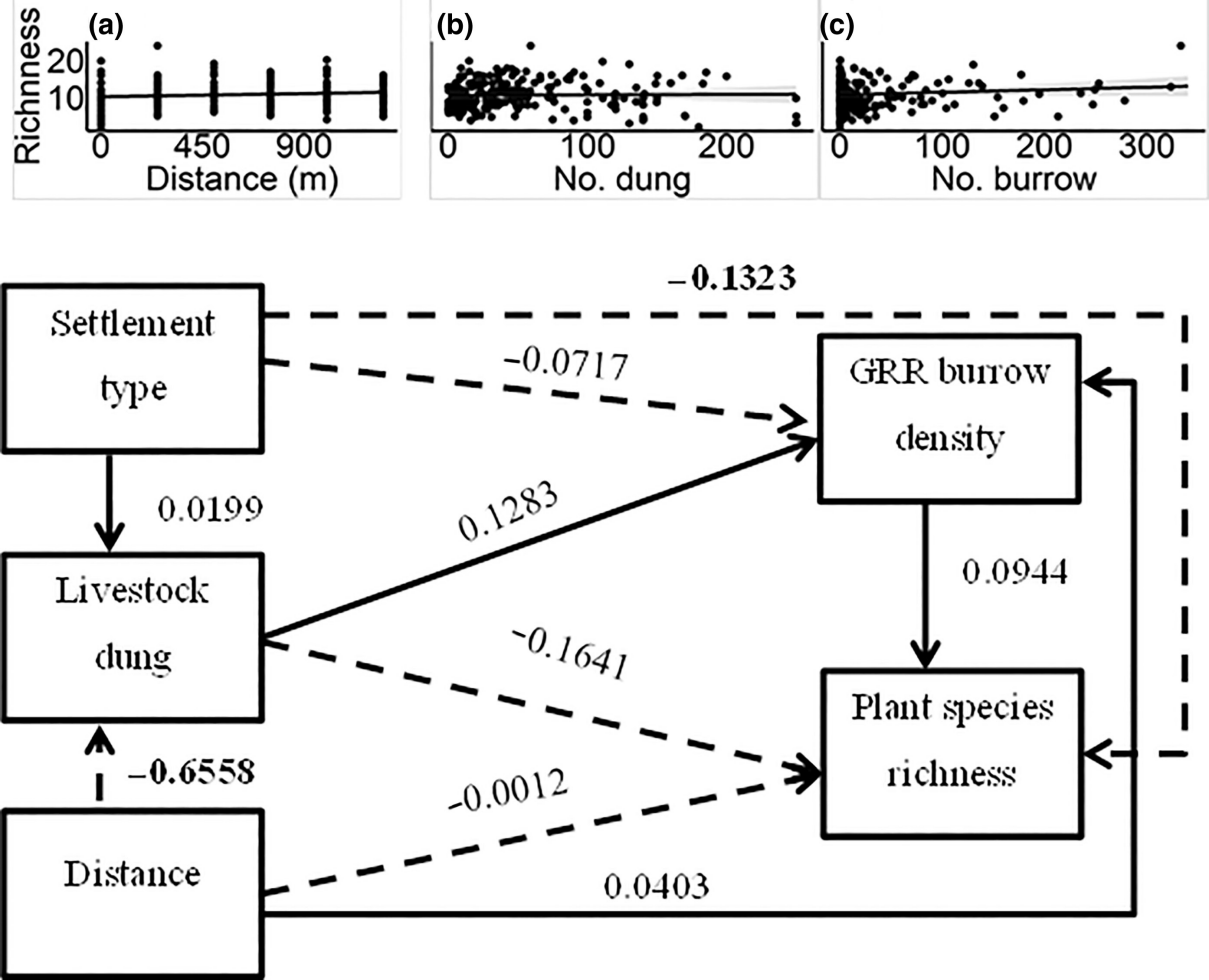
Standardized path coefficients of the direct effects of settlement type (seasonal), distance from settlement, livestock dung abundance and giant root‐rat burrow density on plant species richness. Paths in solid line indicate positive associations, while those with broken line indicate negative associations. Path coefficients indicated in bold font denote statistically significant effect at *p* < .05. The inset figures show the relationships of distance from settlement (a), livestock dung abundance (b) and giant root‐rat (GRR) burrow density (c) with plant species richness.

We used path analyses to simultaneously quantify and test (1) the effects of human settlement type differentiating between traditional seasonal versus permanent, and distance from settlement on livestock grazing intensity; (2) the direct and indirect effects of settlement type, distance from settlement, livestock grazing and vegetation variables (i.e. vegetation cover and plant species richness) on giant root‐rat burrow density; and (3) the potential influences of human activities on the reciprocal effects of giant root‐rat burrow density on the vegetation variables. Thus, to disentangle the effect of vegetation patterns on giant rot‐rat burrow density and vice‐versa, we defined two sets of path models, each consisting of two path models involving one of the two vegetation variables as a predictor or response (Shipley, 2009; for detail see also Figures 2, 3, 4, 5). For each path model, we fitted three multiple regression models using the glmmTMB R package (Brooks et al., 2017). We conducted all analyses in the R environment (R Core Team, 2020) and the full R‐script of all analyses is given in Appendix S1.

We first fitted each regression using generalized linear mixed‐effects models (GLMM), specifying transect nested within site as random variables, to account for their potential effects on the response variables that would not be explained by the fixed‐effect variables. Whenever model convergence problems were detected, we updated such models by changing the default optimizer nlminb() to the BFGS() option from optim() function of the glmmTMB R package (Brooks et al., 2017), which led to model convergences in all cases (Brooks et al., 2017). We then used diagnostic plots in the DHARMa R package (Hartig, 2022) and tested each fitted model for uniformity, dispersion, zero‐inflation, homoscedasticity and outliers. Whenever significant violations in any of these assumptions were detected, we revised model structure and rerun again by adding zero‐inflation and/or dispersion model components, depending on the diagnostic test results (Brooks et al., 2017; Hartig, 2022). This model revision solved the assumption problems detected. Summary of model specification and error distribution used for all finally fitted regression models are provided on Table 1 and briefly described as follows.

**TABLE 1:** Description of regression model structure fitted using glmmTMB for path models analysing the effects of human activities and vegetation variables on giant root‐rat burrow density (a), and the effects of human activities and giant root‐rat burrow density on vegetation variables (b).

Response variable	Regression model structure
(a) Effects of human activities and vegetation variables on giant root‐rat burrow density	
Level 1: Livestock dung abundance	Dung ~ settlement type + distance from settlement + (1|site/transect), family = nbinom2
Level 2(a): Vegetation cover	Vegetation cover ~ settlement type + distance from settlement + dung + (1|site/transect), dispformula = ~ dung, family = beta_family
Level 2(b): Plant species richness	Richness ~ settlement type + distance from settlement + dung + (1|site/transect), dispformula = ~ dung, family = nbinom2
Level 3(a): Giant root‐rat burrow density	Giant root‐rat burrow density ~ settlement type + distance from settlement + dung + vegetation cover + (1|site/transect), ziformula = ~ settlement type + dung + vegetation cover, family = nbinom2
Level 3(b): Giant root‐rat burrow density	Giant root‐rat burrow density ~ settlement type + distance from settlement + dung + plant species richness + (1|site/transect), ziformula = ~ settlement type + dung, family = nbinom2
(b) Effects of human activities and giant root‐rat burrow density on vegetation variables	
Level 1: Livestock dung abundance	Dung ~ settlement type + distance from settlement + (1|site/transect), family = nbinom2
Level 2: Giant root‐rat burrow density	Giant root‐rat burrow density ~ settlement type + distance from settlement + dung + (1|site/transect), ziformula = ~ settlement type, dispformula = ~ dung + settlement type + distance from settlement, family = nbinom2
Level 3(a): Vegetation cover	Vegetation cover ~ settlement type + distance from settlement + dung + giant root‐rat burrow density + (1|site/transect), family = beta_family
Level 3(b): Plant species richness	Richness ~ settlement type + distance from settlement + dung + giant root‐rat burrow density + (1|site/transect), dispformula = ~ dung, family = nbinom2

*Note*: Each regression model consists of fixed effects, random effects—indicated as ‘1|site/transect’, and, where applicable, zero‐inflation and dispersion model components that are incorporated via ziformula and dispformula functions, respectively.

For path models in which giant root‐rat burrow density was a response, the first regression modelled the effects of settlement type and distance from settlement on livestock dung abundance using GLMM, with a negative binomial error distribution in the glmmTMB R package (Brooks et al., 2017). In the second regression, we included settlement type, distance from settlement and livestock dung abundance as fixed effects and either vegetation cover (modelled using beta‐family error distribution) or plant species richness (modelled using negative binomial error distribution) as a response. We fitted these regressions with a dispersion component added to each model. Finally, we analysed the effects on giant root‐rat burrow density of settlement type, distance from settlement, livestock dung density and either vegetation cover or plant species richness, using a zero‐inflated GLMMs (Table 1). For the second set of path models analysing the reciprocal effects of giant root‐rat burrow density on vegetation variables and the influences of human activities on the effects, we reversed path directions between each of the two vegetation variables and giant root‐rat burrow density. Here, we fitted three regression models for each path model and the first regression was identical as for previous path models. The second regression, which was also identical across the two path models in this set, modelled giant root‐rat burrow density as a response using a GLM with negative binomial error distribution, and settlement type, distance from settlement and livestock dung abundance as fixed effects. In the final regression models, we included settlement type, distance from settlement, livestock dung abundance and giant root‐rat burrow density as fixed effects, and either vegetation cover or plant species richness as a response (for details on model specifications see Table 1).

For each final regression model, described above, we checked for multicollinearity among predictors using the ‘performance’ R package (Lüdecke, 2021); this confirmed lack of collinearity problem, in all models collinearity values ranged between 1.00 and 1.67. Thus, we obtained raw (unstandardized) regression coefficients and associated *p*‐values of each predictor, as well as standardized path coefficients, using the ‘MuMIn’ R Package (Bartoṅ, 2022). Finally, we obtained coefficient estimates of the indirect effect of each predictor on the response variable in each path model, as the product of the standardized path coefficients of two sequential paths in a model (Shipley, 2009; for detail on the causal models, see Figures 2, 3, 4, 5). We tested the statistical significance of each indirect effect using the Sobel Test (Soper, 2022). We also obtained conditional and/or marginal *R*‐squared values using the package ‘performance’ (Lüdecke, 2021), to assess the proportion of variation of the response explained by the model.

## 3 RESULTS

We recorded a mean (±SE) number of 53.28 ± 4.21 livestock dung per plot, ranging from 0 to 500 across plots and density of 852.48 ± 67.36 per ha. We counted a mean number of 30.76 ± 4.12 giant root‐rat burrows per plot (range: 0–333 burrows), with a density at 492.16 ± 65.92 burrows per ha. Mean proportion of vegetation cover was 0.43 ± 0.12 (range: 0.00–0.92). We recorded a total of 68 plant species across plots, with a range of 2–24 species and a mean number of 10.57 ± 0.26 of species per plot.

### 3.1 Human activities and their effects on vegetation

The regression models analysing the effects of human settlement type, distance from settlement and livestock grazing intensity on vegetation explained 51%–100% of the total variation in the response variable (Tables 2 and 3). We found a decreased livestock dung abundance with increasing distance from settlement areas (*Z* = −11.189, *p* < .001), and a nonsignificant effect of settlement type on dung abundance (*Z* = 0.302, *p* > .05) (Tables 2 and 3). We found a significantly higher vegetation cover at seasonal human settlement areas compared with that at permanent settlement areas (*Z* = 2.200, *p* < .01; Table 2), while we found decreased vegetation cover with increasing livestock dung abundance (*Z* = −3.222, *p* < .01, Table 2). Although the direct positive effect of distance from settlement on vegetation cover was nonsignificant (Table 2), it had a significant indirect positive effect due to its negative association with livestock dung abundance (Sobel test = 2.644, *p* < .05; Table 4) that in turn also had a negative effect on vegetation cover. Regarding the effects of human activities on plant species richness, we found significantly lower richness at seasonal settlements compared with that at permanent ones (*Z* = −2.068, *p* < .05; Table 3).

**TABLE 2:** Results of regression models analysing the effects of settlement type (seasonal vs. permanent), distance from settlement, livestock dung abundance and vegetation cover on giant root‐rat burrow density.

Variables	Unstd. Est. ± SE	Std. Est.	*Z*‐value
Level 1: Abundance of livestock dung (Conditional/marginal *R* ^2^: .51/.32)			
Intercept	4.5638 ± 0.2025		22.533***
Settlement type (seasonal)	0.0340 ± 0.1127	0.0199	0.302
Distance from settlement	−0.0014 ± 0.0001	−0.6558	−11.189***
Level 2: Vegetation cover (Conditional/marginal *R* ^2^: 1.00/.64)			
Intercept	−0.3309 ± 0.1514		−2.186*
Settlement type (seasonal)	0.2544 ± 0.1156	0.2165	2.200*
Distance from settlement	0.0002 ± 0.0001	0.1453	1.488
Livestock dung abundance	−0.0045 ± 0.0014	−0.4149	−3.222**
Level 3: Giant root‐rat burrow density (Conditional/marginal *R* ^2^: .23/.11)			
Intercept	4.3281 ± 0.5002		8.654***
Settlement type (seasonal)	−0.3546 ± 0.2363	−0.0885	−1.501
Distance from settlement	0.0003 ± 0.0003	0.0622	0.933
Livestock dung abundance	0.0047 ± 0.0038	0.1266	1.219
Vegetation cover	−1.8808 ± 0.6132	−0.1671	−3.067**

*Note*: Given are unstandardized regression coefficients and their standard errors (Unstd. Est. ± SE), standardized path coefficients (Std. Est.), and marginal/conditional *R* ^2^ values. *Z*‐values with asterisk indicate significant effects at levels of *p < .05, **p < .01, ***p < .001.

**TABLE 3:** Results of regression models analysing the direct effects of settlement type (seasonal vs. permanent), distance from settlement, livestock dung abundance (proxy for grazing intensity) and plant species richness on giant root‐rat burrow density.

Variables	Unstd. Est. ± SE	Std. Est.	*Z*‐value
Level 1: Abundance of livestock dung (Conditional/marginal *R* ^2^: .51/.32)			
Intercept	4.5638 ± 0.2025		22.533***
Settlement type (seasonal)	0.0340 ± 0.1127	0.0199	0.302
Distance from settlement	−0.0014 ± 0.0001	−0.6558	−11.189***
Level 2: Plant species richness (Conditional/marginal *R* ^2^: 1.00/.10)			
Intercept	2.4340 ± 0.1118		21.768***
Settlement type (seasonal)	−0.0884 ± 0.0434	−0.0686	−2.038*
Distance from settlement	0.00001 ± 0.00001	0.0501	0.040
Livestock dung abundance	−0.0010 ± 0.0006	0.0755	−1.605
Level 3: Giant root‐rat burrow density (Conditional/marginal *R* ^2^: .24/.05)			
Intercept	3.2425 ± 0.5977		5.425***
Settlement type (seasonal)	−0.3524 ± 0.2816	−0.1387	−1.252
Distance from settlement	0.0002 ± 0.0003	0.0734	0.675
Livestock dung abundance	0.0043 ± 0.0039	0.1828	1.091
Plant species richness	0.0306 ± 0.0341	0.0931	0.898

*Note*: Given are unstandardized regression coefficients and standard errors (Unstd. Est. ± SE), and standardized coefficients (Std. Est.), *Z*‐values, and marginal/conditional *R* ^2^ values of each regression model depending on if random effects were included in the models (see Section 2 for details). *Z*‐values with asterisk indicate significant effects at levels of *p < .05, **p < .01, ***p < .001.

**TABLE 4:** Estimated coefficients of indirect effects (IE) on giant root‐rat burrow density of settlement type (seasonal) and distance from settlement (Distance) via livestock dung abundance and vegetation variables, and of livestock dung abundance via vegetation variables.

Predictor	Mediator	Response	IE	Sobel test statistic
Path model 1: Effects of human activities and vegetation cover on giant root‐rat burrow density				
Settlement type	Livestock dung	Vegetation cover	−0.0083	−0.3
Distance	Livestock dung	Vegetation cover	0.2721	2.644*
Settlement type	Livestock dung	Giant root‐rat burrow density	0.0013	−0.302
Distance	Livestock dung	Giant root‐rat burrow density	−0.0445	−2.056*
Settlement type	Vegetation cover	Giant root‐rat burrow density	−0.0139	−2.211*
Distance	Vegetation cover	Giant root‐rat burrow density	−0.0093	−1.387
Livestock dung	Vegetation cover	Giant root‐rat burrow density	0.0266	2.165*
Path model 2: Effects of human activities and plant species richness on giant root‐rat burrow density				
Settlement type	Livestock dung	Plant species richness	0.0015	0.295
Distance	Livestock dung	Plant species richness	−0.0495	−1.412
Settlement type	Livestock dung	Giant root‐rat burrow density	0.0036	0.291
Distance	Livestock dung	Giant root‐rat burrow density	−0.1199	−1.099
Settlement type	Plant species richness	Giant root‐rat burrow density	−0.0064	−0.825
Distance	Plant species richness	Giant root‐rat burrow density	0.0047	0.736
Livestock dung	Plant species richness	Giant root‐rat burrow density	0.0070	0.758

* *p* < .05.

### 3.2 Effects of human activities on giant root‐rat burrow density

The regression model in the path models analysing the effects of human settlement, livestock grazing and vegetation on giant root‐rat burrow density explained 23%–24% of the total variation in the response variables (Tables 2 and 3). Considering the path model examining the effects of settlement, livestock dung abundance and vegetation cover on giant root‐rat burrow density, a significant direct effect was found only for increasing vegetation cover that led to decreased root‐rat burrow density (*Z* = −3.222, *p* < .01). We found that increasing livestock dung abundance and indirectly via vegetation cover (Sobel test = 2.165, *p* < .001; Table 4; Figure 2) led to increased giant root‐rat burrow density (*Z* = 2.096, *p* < .05; Table 2). Seasonal settlement type (Sobel test = −2.211, *p* < .05), compared with permanent settlement type, and distance from settlement (Sobel test = −1.387, *p* < .05) had significant indirect negative effects on giant root‐rat burrow density, both via vegetation cover (Table 4). Increasing livestock dung abundance resulted in increased vegetation cover that in turn resulted in increased giant root‐rat burrow density (Sobel test = 2.165). In the second path model including plant species richness, we did not find a significant direct and/or indirect effect of settlement type, distance from settlement, livestock dung abundance or plant species richness on giant root‐rat burrow density (Tables 3 and 4; Figure 3).

### 3.3 Influences of human activities on reciprocal effects of giant root‐rat on vegetation

In the path models analysing the reciprocal effects of giant root‐rat on vegetation, regression models explained 21%–100% of the total variations in the response variables (Tables 5). Examining the reciprocal effect of giant root‐rat burrow density on vegetation cover, we found a significantly decreased vegetation cover with increasing giant root‐rat burrow density (*Z* = −2.605, *p* < .05; Table 5; Figure 4). We also found higher vegetation cover at the seasonal settlement type, compared with the permanent settlement type, increased vegetation cover with increasing distance from settlement (Table 5). Considering the reciprocal effect of giant root‐rat burrow density on plant species richness, we neither found a significant direct effect of giant root‐rat burrow density on plant species richness, nor of the direct or indirect effect of settlement type, distance from settlement, or livestock dung abundance (Tables 5 and 6; Figure 5).

**TABLE 5:** Results of path models analysing the effects of giant root‐rat burrow density on plant species richness and vegetation cover.

Variables	Unstd. Est. ± SE	Std. Est.	*Z*‐value
Level 1: Livestock dung abundance (Conditional/marginal *R* ^2^: .51/.32)			
Intercept	4.5638 ± 0.2025		22.533***
Settlement type (seasonal)	0.0340 ± 0.1127	0.0199	0.302
Distance from settlement	−0.0014 ± 0.0001	−0.6558	−11.189***
Level 2: Giant root‐rat burrow density (Conditional/marginal *R* ^2^: .21/.05)			
Intercept	3.4871 ± 0.5355		6.512***
Settlement type (seasonal)	−0.3445 ± 0.2974	−0.0717	−1.158
Distance from settlement	0.0002 ± 0.0003	0.0403	0.710
Livestock dung abundance	0.0057 ± 0.0054	0.1283	1.050
Level 3(a): Vegetation cover (Conditional/marginal *R* ^2^: .86/.72)			
Intercept	−0.3332 ± 0.1647		−2.022*
Settlement type (seasonal)	0.2671 ± 0.1015	0.1671	2.631**
Distance from settlement	0.0004 ± 0.0001	0.1878	2.486*
Livestock dung abundance	−0.0022 ± 0.0012	−0.3821	−4.700***
Giant root‐rat burrow density	−0.0022 ± 0.0009	−0.1674	−2.605**
Level 3(b): Plant species richness (Conditional/marginal *R* ^2^: 1.00/.12)			
Intercept	2.4230 ± 0.1119		21.644***
Settlement type (seasonal)	−0.0933 ± 0.0434	−0.1323	−2.152*
Distance from settlement	−0.0001 ± 0.0006	−0.0012	−0.015
Livestock dung abundance	−0.0011 ± 0.0006	−0.1641	−1.702
Giant root‐rat burrow density	0.0006 ± 0.0004	0.0944	1.566

*Note*: Given for each regression model are conditional/marginal *R* ^2^ values depending on if random effects were included in the models (see Section 2 for details) and values of unstandardized regression coefficients and their standard errors (Unstd. Est. ± SE), and standardized path coefficients (Std. Est.). *Z*‐values with asterisk indicate significant effects at significance levels of *p < .05, **p < .01, ***p < .001.

**TABLE 6:** Estimated coefficients of indirect effects (IE) on plant species richness and vegetation cover of settlement type (seasonal) and distance from settlement (Distance) via livestock dung abundance and giant root‐rat burrow density, and of livestock dung abundance via giant root‐rat burrow density.

Predictor	Mediator	Response	IE	Sobel test statistic
Path model 1: Effects of human activities and giant root‐rat burrow density on vegetation cover				
Settlement type	Livestock dung	Vegetation cover	−0.0076	−0.298
Distance	Livestock dung	Vegetation cover	0.2506	1.818*
Settlement type	Giant root‐rat burrow density	Vegetation cover	0.0012	1.047
Distance	Giant root‐rat burrow density	Vegetation cover	−0.0067	−0.643
Livestock dung	Giant root‐rat burrow density	Vegetation cover	−0.0215	−0.969
Path model 2: Effects of human activities and giant root‐rat burrow density on plant species richness				
Settlement type	Livestock dung	Plant species richness	−0.0033	−0.298
Distance	Livestock dung	Plant species richness	0.1076	1.218
Settlement type	Giant root‐rat burrow density	Plant species richness	−0.0068	−0.917
Distance	Giant root‐rat burrow density	Plant species richness	0.0038	0.609
Livestock dung	Giant root‐rat burrow density	Plant species richness	0.0121	0.863

* *p* < .05.

## 4 DISCUSSION

Our results demonstrate that giant root‐rat burrow density and vegetation cover reciprocally affect each other and are modulated by human activities. In line with our predictions, we found increased livestock dung abundance with decreasing distance from settlement, suggesting heavier grazing intensity near settlements. Increasing livestock dung abundance in turn led to decreased vegetation cover, and indirectly via vegetation cover led to increased giant root‐rat burrow density. This positive association of giant root‐rat burrow density with livestock grazing intensity and decreasing distance to human settlement sites is an interesting finding of our study that revealed the root‐rat's synanthropic association, a phenomenon that has not been well‐studied, although previous research has indicated a potential synanthropic association (Ossendorf et al., 2019) as the root‐rats are known to prefer sites with lower vegetation cover (Asefa et al., 2022; Šklíba et al., 2017; Tallents, 2007). Aligning with previous research, our results indicate giant root‐rats reciprocally negatively affected vegetation cover, with human activities modulating this reciprocal association.

Our finding of increased giant root‐rat burrow density with livestock grazing intensity, indirectly via reduced vegetation cover, is in line with results of our recent independent study on giant root‐rats (Asefa et al., 2022). This finding suggests that livestock grazing‐induced decreases in vegetation cover potentially benefit habitat occupancy of subterranean small rodents, including giant root‐rats that are adapted to life in grassland ecosystems (Šumbera et al., 2018). In fact, giant root‐rats are found to be very abundant even in heavily degraded areas caused by livestock grazing around settlement areas (Šklíba et al., 2017; Tallents, 2007). Similar studies on other subterranean rodents, such as the Plateau zokor (*Myospalax baileyi*) in the Chinese Tibetan Plateau (Wang et al., 2019, 2020) and black‐tailed prairie dogs (*Cijnomys ludovicianus*, Knowles 1986), have also reported positive effects of livestock grazing on rodents. Besides this positive effect of livestock grazing on giant root‐rat habitat, it is also possible that livestock grazing affects nutrient cycling, by increasing the availability of nitrogen for rapid regrowth of the grazed plants, which results in increased biomass of young, palatable plant tissues (Hobbs, 1996; Tallents, 2007). This can improve the quality of food for herbivorous rodents, such as giant root‐rats. Despite this, some other studies showed that heavy livestock grazing negatively impacts a marsupial ecosystem engineer (Eldridge & Soliveres, 2023; Neilly & Schwarzkopf, 2018).

Consistent with our second prediction, livestock dung abundance also appeared to modulate the association of distance from settlement with giant root‐rat burrow density, as shown by the indirect, via livestock dung abundance, negative effect of distance from settlement on giant root‐rat burrow density (Table 4). This result is a consequence of heavier livestock grazing intensity around settlement areas, thereby reducing vegetation cover and creating open habitat for giant root‐rats and other rodents (see also Reitalu et al., 2010). This association of giant root‐rats with human settlement appears to be stronger at permanent settlement areas, as shown in our finding of decreased giant root‐rat burrow density at seasonal settlement sites due to higher vegetation cover at seasonal than at permanent settlements (Tables 2 and 4). The fostering effects of settlement abandonment on vegetation cover have also been reported in many studies elsewhere (e.g. Mayer et al., 2019; Pavlů et al., 2019) and can be attributed to colonization of grazing‐induced degraded areas by disturbance‐tolerant plant species that exploit abundantly available resources and to relaxation from damages due to temporary grazing abandonment (Bokdam, 2001; Niu et al., 2019). Our above findings highlight the presence of a synanthropic association of giant root‐rats, which has not been revealed prior to this study. Our findings of an overall positive effect of livestock grazing on giant root‐rat have to be interpreted with caution for three main reasons. First, our study did not consider the potential effects of sheep and goats, which are reported to affect subterranean rodents differently, mainly via browsing, to that of large‐sized livestock (cattle and horses) (Wang et al., 2019, 2020). Second, our study period encompassed only the dry season. However, since food abundance both for livestock and giant root‐rat is higher during the wet season, grazing intensity and its effects on giant root‐rat burrow density may differ in the wet season (Šklíba et al., 2017; Vial et al., 2011), which likely is weaker association in the wet season as more food available. In addition, Šklíba et al. (2017) have found that giant root‐rats show a slight differences in their mobility between dry and wet seasons, their engineering activities and impacts can also differ between wet season. And, third, habitat modification and degradation due to livestock overgrazing have been considered as the major threat to the giant root‐rat (BMNP, 2017; Lavrenchenko & Kennerley, 2016). This may suggest that the positive association of giant root‐rats with livestock grazing intensity we found, as also reported by Šklíba et al. (2017), may not necessarily mean that grazing is always beneficial to giant root‐rats, rather may suggest giant root‐rats' reliance on underground parts of plants as a food source where aboveground vegetation is degraded (for detail on feeding habit of giant root‐rat, see Yaba et al., 2011). Thus, it seems that livestock grazing is likely a detrimental threat to survival of giant root‐rats when the impacts involve both aboveground and belowground vegetation biomass. As such, livestock density matters in shaping root‐rat populations and their co‐occurrence may clearly depend on the management of livestock density. In order to avoid the potential negative impacts of the currently growing unregulated grazing practices in the Bale Mountains on the giant root‐rats and other co‐occurring endemic rodents, planning and implementation of grazing management policy should rely on understanding of thresholds of grazing level beneficial and tolerable to giant root‐rats. In the path model including species richness as a predictor, the lack of significant effect of plant species richness on giant root‐rat burrow density contradicts our expectation and the positive relationship found in our recent study on giant root‐rat (Asefa et al., 2022). The discrepancy in results of these studies seem to be due to differences in the sampling approach, more wetland habitats were sampled in the previous study which in turn was positively associated with higher plant species richness (Asefa et al., 2022). Yet, we believe that our present finding is more plausible; particularly, considering the food generalist behaviour of the giant root‐rats (Beyene, 1986; Yaba et al., 2011), plant species richness may play minimal role in determining the rodents' distribution.

Analysing the reciprocal effects of giant root‐rat burrow density on vegetation variables and the effects of human activities, our findings showed that increasing giant root‐rat burrow density led to decreased vegetation cover. This finding is consistent with the well‐known negative effects of subterranean rodents on vegetation cover (Asefa et al., 2022; Beyene, 1986; Haussmann, 2017; Šklíba et al., 2017; Wu et al., 2015; see also Valkó et al., 2021 for a similar species, the Steppe Marmot (*Marmota bobak*)) and is attributed to vegetation biomass removal by giant root‐rat's bioturbation and direct feeding. Through this reciprocal effect, giant root‐rats' engineering does not only affect vegetation but also positively affects the giant root‐rats themselves, because the reduction in vegetation cover they cause eventually creates higher quality habitat. This supposition holds true, in the light of findings of previous studies (Miehe & Miehe, 1994; Šklíba et al., 2017; Wraase et al., 2022; Yalden, 1975) that giant root‐rats' own long‐term burrowing activity plays an important role in their habitat selection. Despite the significant effects of giant root‐rat burrow density on vegetation cover, we found a nonsignificant effect on plant species richness, which is in line with finding of our previous study on giant root‐rat (Asefa et al., 2022), as well as studies on other rodents elsewhere (e.g. Wu et al., 2015).

Overall, our results showed that vegetation and giant root‐rat reciprocally affect each other and human activities related to settlement and livestock grazing influence these natural reciprocal relationships, which likely hold true for other subterranean rodents. Here, human activities interactively caused decreased vegetation cover that in turn led to increased giant root‐rat burrow density, although these effects are more pronounced at permanent settlements. Reciprocally, giant root‐rat engineering is found to further reduce vegetation cover that has continuously been affected by human activities, thereby enhancing their habitat suitability (Šklíba et al., 2017; Wraase et al., 2022). These results highlight that giant root‐rats play a synanthropic ecological engineering role in shaping vegetation cover by reducing cover but leading to increased cover after abandoning their burrows (see Šklíba et al., 2017); thereby contributing to our understanding of the effects of subterranean burrowing herbivore animals on ecosystem structure and processes in the face of growing biodiversity loss due to global (e.g. climate change) and local (e.g. human activities) change drivers. Despite this, results of this study should be interpreted cautiously, because we considered only present engineering activity of giant root‐rats. Similar to many subterranean engineer rodents across the globe (e.g. Davidson et al., 2012; Jones et al., 1997; Reichman & Seabloom, 2002), giant root‐rats create a mosaic of sites differing in the age of the engineered burrow marks, which are known to differ in vegetation and soil characteristics (Šklíba et al., 2017). Contrary to the negative effects of present engineering we found in our study, past engineering activities of subterranean rodents are often known to positively affect vegetation cover and plant species richness due to colonization by plant species of new spaces and nutrient rich microhabitats created at old, abandoned rodent burrows (Šklíba et al., 2017; Zhang & Liu, 2003). This implies that present and past engineering activities of rodents, including giant root‐rats, can have antagonistic or opposite effects on vegetation variables and soil properties (Šklíba et al., 2017; Zhang & Liu, 2003), a mechanism through which ecological engineering rodents likely lead to stable and resilient ecosystem structure and processes (see Jones et al., 1997). Thus, further research should focus on investigating the effects of human activities and giant root‐rat past and present engineering activities on vegetation, including plant functional trait composition.

## AUTHOR CONTRIBUTIONS

**Addisu Asefa:** Conceptualization (supporting); formal analysis (lead); investigation (lead); methodology (equal); validation (equal); visualization (lead); writing – original draft (lead); writing – review and editing (equal). **Victoria Reuber:** Conceptualization (supporting); formal analysis (supporting); visualization (supporting); writing – review and editing (equal). **Georg Miehe:** Conceptualization (lead); validation (equal); writing – review and editing (equal). **Luise Wraase:** Conceptualization (supporting); validation (equal); writing – review and editing (equal). **Tilaye Wube:** Conceptualization (supporting); validation (equal); writing – review and editing (equal). **Dana G. Schabo:** Conceptualization (lead); formal analysis (supporting); methodology (equal); validation (equal); writing – review and editing (equal). **Nina Farwig:** Conceptualization (lead); formal analysis (supporting); methodology (equal); validation (equal); writing – review and editing (equal).

## FUNDING INFORMATION

This work was supported by the German Research Council (DFG) within the Research Unit 2358 (‘The Mountain Exile Hypothesis’) [NA 783/12‐2, FA‐925/14‐1 und SCHA‐2085/3‐1, MI271/33‐2].

## CONFLICT OF INTEREST STATEMENT

The authors have no conflict of interest to declare.

## Supporting information


Appendix S1

## Data Availability

The raw data and R code used in this study are located in The Dryad Data Platform repository, available at https://doi.org/10.5061/dryad.cc2fqz6bx.
