# Supplementary material for: Human activities modulate reciprocal effects of a subterranean ecological engineer rodent, Tachyoryctes macrocephalus, on Afroalpine vegetation cover
Source: Ecol Evol. 2023 Jul 17;13(7):e10337. doi: 10.1002/ece3.10337 (PMC10350814; doi:10.1002/ece3.10337)
Supplement: Supplementary file 1 — Appendix S1 [file ECE3-13-e10337-s001.docx]

Rscript

#### RScript used to analyze the direct and indirect, via vegetation, effects of human activities on giant root-rat and reciprocal effects of the rat on vegetation

#Manuscript: Asefa et al. Human activities modulate reciprocal feedback effects of a subterranean ecological engineer rodent on Afroalpine vegetation

#Load packages

library(glmmTMB)

library(TMB)

library(semEff)

library(lme4)

library(effects)

library(multcomp)

library(dplyr)

library(Hmisc)

library(corrplot)

library(ggpubr)

library(MuMIn)

library(performance)

library(DHARMa)

library(dplyr)

library(ggeffects)

library(car)

library(boot)

library(gsl)

library(parallel)

library(MASS)

library(stats)

library(utils)

library(knitr)

library(markdown)

library(rmarkdown)

library(DiagrammeR)

library(emmeans)

library(igraph)

library(methods)

library(nlme)

library(randomForest)

library(multcompView)

library(devtools)

library(ggplot2)

library(betareg)

######################################################

#Description of data and variables

#All variables used in the analysis are stored in the file GRR2021v1F and defined as follows:

#Data used for the analyses were collected from 214 plots established along 36 transects established at 6 sites site

#The following are variable abbreviation as used in the modelling

#settype: settlement type (permanent vs seasonal)

#distance: distance of a sample plot from a settlement area

#dung: number of livestock dung counted within 25 x 25m plot

#freshholes: number of giant root-rat fresh burrows counted within 25x25m plot

#S: plant species richness recorded within a plot

#vegcvrall: vegetation cover within a plot

#Level 1: examine the effects of settlement type and distance on cow dung

#Level 1: effects of settlement TYPE AND DISTANCE FROM SETTLEMENT on grazing INTENSITY

#1.1: fit with random effect component

dunghnbm1 = glmmTMB(dung~ settype+distance + (1|site/transect),

data=GRR2021v1F, family=nbinom2,

na.action = na.fail) #converged

testDispersion(dunghnbm1) #dispersion is fine

testZeroInflation(dunghnbm1) #No problem detected

simulationhrp1sA11 <- simulateResiduals(fittedModel = dunghnbm1, plot = F)

plot(simulationhrp1sA11) #Quantile deviation detected

plotResiduals(simulationhrp1sA11, form = GRR2021v1F$settype) #Fine

plotResiduals(simulationhrp1sA11, form = GRR2021v1F$distance)#Levene test for homogeneity of variance significant

summary(dunghnbm1)

r2(dunghnbm1)

#1.2: fit without random effect

dunghnbm2 = glmmTMB(dung~ settype+distance,

data=GRR2021v1F, family=nbinom2) #

testDispersion(dunghnbm2) #dispersion is fine

testZeroInflation(dunghnbm2) #No problem detected

simulationhrp1sA12 <- simulateResiduals(fittedModel = dunghnbm2, plot = F)

plot(simulationhrp1sA12) #Quantile deviation detected

plotResiduals(simulationhrp1sA12, form = GRR2021v1F$settype) #Fine

plotResiduals(simulationhrp1sA12, form = GRR2021v1F$distance)#Levene test for homogeneity of variance significant

summary(dunghnbm2)

r2(dunghnbm2)

#Reran by including zi formula

dunghnbm3 = glmmTMB(dung~ settype+distance + (1|site/transect) ,

ziformula = ~1, data=GRR2021v1F, family=nbinom2)

testDispersion(dunghnbm3) #dispersion is fine

testZeroInflation(dunghnbm3) #No problem detected

simulationhrp1sA13 <- simulateResiduals(fittedModel = dunghnbm3, plot = F)

plot(simulationhrp1sA13)#plot(simulationS_norm) #quanltile deviation detected

plotResiduals(simulationhrp1sA13, form = GRR2021v1F$settype)#no problem

plotResiduals(simulationhrp1sA13, form = GRR2021v1F$distance)#quantile deviation detected

summary(dunghnbm3)

r2(dunghnbm13)

#############################

#Decision: model #dunghnbm1 - AIC = 2053.6, R2 = 39; #dungnbm2 - AIC = 2082.0, R2 = 27.7; #dungnbm3: AIC = 2052, R2 = 29.5

#use #dunghnbm1 as best fit and extract parameter estimates

print(R2_dunghnbm1)

R2_dunghnbm1 <- cor(GRR2021v1F$dung, predict(dunghnbm1, type = "response"))^2

sd.yhat_dunghnbm1 <- sqrt(var(predict(dunghnbm1, type = "link"))/R2_dunghnbm1) # according to Lefcheck, this approach is also

beta_sd_dungsttla <-fixef(dunghnbm1)[[1]][2] * sd(as.numeric(GRR2021v1F$settype)[-1L])/sd.yhat_dunghnbm1

beta_sd_dungdista <-fixef(dunghnbm1)[[1]][3] * sd(GRR2021v1F$distance)/sd.yhat_dunghnbm1

r2(dunghnbm1)

print(beta_sd_grazingsettl)

print(beta_sd_grazingdist)

model_performance(dunghnbm1, metrics = "all")

MuMIn::dredge(dunghnbm1)

#############################

#LEVEL 2: EXAMINE EFFECTS of settlement, distance and grazing ON VEGETATION

#Level 2A: fit the effects of settlement type, distance and grazing on vegetation cover

#Fit with random component

vegcvrallm1 = glmmTMB(vegcvrall~settype+distance + dung +(1|site/transect),

data=GRR2021v1F,

family=beta_family(),

na.action = na.fail) #did not converge

#Fit without random effect

vegcvrallm2 = glmmTMB(vegcvrall~settype+distance + dung ,

data=GRR2021v1F,

family=beta_family(),

na.action = na.fail) #converged #best fit

testDispersion(vegcvrallm2) #dispersion is fine

testZeroInflation(vegcvrallm2) #No problem detected

simulationvegcv2 <- simulateResiduals(fittedModel = vegcvrallm2, plot = F)

plot(simulationvegcv2)#quantile deviation detected

plotResiduals(simulationvegcv2, form = GRR2021v1F$settype)

plotResiduals(simulationvegcv2, form = GRR2021v1F$distance) #within-group deviation detected

plotResiduals(simulationvegcv2, form = GRR2021v1F$dung)#quantile deviation detected

summary(vegcvrallm2)

r2(vegcvrallm2)

#Refit by including dung as a dispersion

vegcvrallm3 = glmmTMB(vegcvrall~settype+distance + dung ,

dispformula = ~dung,

data=GRR2021v1F,

family=beta_family(),

na.action = na.fail) #converged #best fit

testDispersion(vegcvrallm3) #dispersion is fine

testZeroInflation(vegcvrallm3) #No problem detected

simulationvegcv2 <- simulateResiduals(fittedModel = vegcvrallm3, plot = F)

plot(simulationvegcv2)#Fine

plotResiduals(simulationvegcv2, form = GRR2021v1F$settype)

plotResiduals(simulationvegcv2, form = GRR2021v1F$distance)

plotResiduals(simulationvegcv2, form = GRR2021v1F$dung)#Fine

summary(vegcvrallm3)

r2(vegcvrallm3)

######

#refit model vegcvrallm3 with random-effect

vegcvrallm3RE = glmmTMB(vegcvrall~settype+distance + dung +(1|site/transect),

dispformula = ~dung,

data=GRR2021v1F,

family=beta_family(),

na.action = na.fail) #did not converge

#Reran model vegcvrallm3RE using BFGS optimizer

vegcvrallm3RE_optim <- update(vegcvrallm3RE,

control=glmmTMBControl(optimizer=optim,

optArgs=list(method="BFGS"))) #model converged

testDispersion(vegcvrallm3RE_optim) #dispersion is fine

testZeroInflation(vegcvrallm3RE_optim) #No problem detected

simulationhrp1sM3REOptim <- simulateResiduals(fittedModel = vegcvrallm3RE_optim, plot = F)

plot(simulationhrp1sM3REOptim)#fine

plotResiduals(simulationhrp1sM3REOptim, form = GRR2021v1F$settype) #Ok

plotResiduals(simulationhrp1sM3REOptim, form = GRR2021v1F$distance) #within-group deviations detected

plotResiduals(simulationhrp1sM3REOptim, form = GRR2021v1F$dung) #Ok

check_collinearity(vegcvrallm3RE_optim)

#CONCLUSION: USE MODEL #vegcvrallm3RE_optim AS BEST FIT FOR PARAMETER ESSTIMATION

#Obtain unstanderdized estimates

summary(vegcvrallm3RE_optim)

r2(vegcvrallm3RE_optim)

R2_vegc21optim <- cor(GRR2021v1F$vegcvrall, predict(vegcvrallm3RE_optim, type = "response"))^2

sd.yhat_vegc1optim <- sqrt(var(predict(vegcvrallm3RE_optim, type = "link"))/R2_vegc21optim) # according to Lefcheck, this approach is also

beta_sd_vegsttlaoptim <-fixef(vegcvrallm3RE_optim)[[1]][2] * sd(as.numeric(GRR2021v1F$settype)[-1L])/sd.yhat_vegc1optim

beta_sd_vegdistaoptim <-fixef(vegcvrallm3RE_optim)[[1]][3] * sd(GRR2021v1F$distance)/sd.yhat_vegc1optim

beta_sd_vegdungoptim1 <-fixef(vegcvrallm3RE_optim)[[1]][4] * sd(GRR2021v1F$dung)/sd.yhat_vegc1optim

#Get standerdized coefficient estimates

beta_sdvegallc1optim <- c(beta_sd_vegsttlaoptim, beta_sd_vegdistaoptim, beta_sd_vegdungoptim1)

print(beta_sdvegallc1optim)

################

str()

#LEVEL 2B: fit the effects of settlement and grazing on plant species richness

#2A.1: fit with random component

hnbmHB1 = glmmTMB(S~settype+distance + dung + (1|site/transect), #Did not converge

data=GRR2021v1F, family=nbinom2())

#Test model assumptions

testDispersion(ShnbmHB2) #dispersion is fine

testZeroInflation(ShnbmHB2) #No problem detected

simulationhrp1sA34 <- simulateResiduals(fittedModel = ShnbmHB2, plot = F)

plot(simulationhrp1sA34)#fine

plotResiduals(simulationhrp1sA34, form = GRR2021v1F$settype)

plotResiduals(simulationhrp1sA34, form = GRR2021v1F$distance) #fine

plotResiduals(simulationhrp1sA34, form = GRR2021v1F$dung) #quantile deviation detected

#Test model assumptions

testDispersion(hnbmHB12023) #dispersion is fine

testZeroInflation(hnbmHB12023) #No problem detected

simulationhrp12023 <- simulateResiduals(fittedModel = hnbmHB12023, plot = F)

plot(simulationhrp12023)#quantile deviations detected

plotResiduals(simulationhrp12023, form = GRR2021v1F$settype) #no problem

plotResiduals(simulationhrp12023, form = GRR2021v1F$distance) #fine

plotResiduals(simulationhrp12023, form = GRR2021v1F$dung) #quantile deviation detected

summary(hnbmHB12023)

r2(hnbmHB12023)

R2S <- r2(hnbmHB12023, ci = NULL, tolerance = 1e-10)

print(R2S)

R2S2 <- r2_mckelvey(hnbmHB12023)

R2S4 <- r2_nakagawa(

hnbmHB12023,

by_group = FALSE,

tolerance = 1e-20)

print(R2S4)

check_singularity(hnbmHB12023, tolerance = 1e-10)

#rerun model hnbmHB12023 using gaussian distribution

hnbmHB12023G = glmmTMB(S~settype+distance + dung + (1|site/transect), #Model did not fit

dispformula = ~dung+settype+distance,

data=GRR2021v1F, family=gaussian())

#Test model assumptions

testDispersion(hnbmHB12023G) #dispersion is fine

testZeroInflation(hnbmHB12023G) #No problem detected

simulationhrp12023G <- simulateResiduals(fittedModel = hnbmHB12023G, plot = F)

plot(simulationhrp12023G)#quantile deviations detected

plotResiduals(simulationhrp12023G, form = GRR2021v1F$settype) #no problem

plotResiduals(simulationhrp12023G, form = GRR2021v1F$distance) #fine

plotResiduals(simulationhrp12023G, form = GRR2021v1F$dung) #quantile deviation detected

summary(hnbmHB12023G)

r2(hnbmHB12023G)

r.squaredLR(hnbmHB12023G)

ShnbmHB3 = glmmTMB(S~settype+distance + dung ,

dispformula=~dung,

data=GRR2021v1F, family=nbinom2)

testDispersion(ShnbmHB3) #dispersion is fine

testZeroInflation(ShnbmHB3) #No problem detected

simulationhrp1sA3Aa <- simulateResiduals(fittedModel = ShnbmHB3, plot = F)

plot(simulationhrp1sA3Aa)#fine

plotResiduals(simulationhrp1sA3Aa, form = GRR2021v1F$settype) #fine

plotResiduals(simulationhrp1sA3Aa, form = GRR2021v1F$distance) #within group deviations detected

plotResiduals(simulationhrp1sA3Aa, form = GRR2021v1F$dung) #Quantile deviations detected

#Change distribution family to gaussian

ShnbmHB4 = glmmTMB(S~settype+distance + dung ,

dispformula = ~ dung ,

data=GRR2021v1F,family=gaussian,

na.action = na.fail)

testDispersion(ShnbmHB4) #dispersion is fine

testZeroInflation(ShnbmHB4) #No problem detected

simulationhrp1sA3a <- simulateResiduals(fittedModel = ShnbmHB4, plot = F)

plot(simulationhrp1sA3a)#plot(simulationS_norm) #fine

plotResiduals(simulationhrp1sA3a, form = GRR2021v1F$settype)

plotResiduals(simulationhrp1sA3a, form = GRR2021v1F$distance)#quantile deviations detected

plotResiduals(simulationhrp1sA3a, form = GRR2021v1F$dung)#quantile deviations detected

summary(ShnbmHB4)

r2(ShnbmHB4)

#Conclusion

#Get parameter estimates for model #ShnbmHB4

print(R2_dunghnbm1)

R2_S104a <- cor(GRR2021v1F$S, predict(ShnbmHB4, type = "response"))^2

sd.yhat_S104a <- sqrt(var(predict(ShnbmHB4, type = "link"))/R2_S104a) # according to Lefcheck, this approach is also

beta_sd_Ssttla1 <-fixef(ShnbmHB4)[[1]][2] * sd(as.numeric(GRR2021v1F$settype)[-1L])/sd.yhat_S104a

beta_sd_Sdista1 <-fixef(ShnbmHB4)[[1]][3] * sd(GRR2021v1F$distance)/sd.yhat_S104a

beta_sd_sdung11 <-fixef(ShnbmHB4)[[1]][4] * sd(GRR2021v1F$dung)/sd.yhat_S104a

#Get standerdized coefficient estimates

check_collinearity(ShnbmHB4)

betaSD_S104a <- c(beta_sd_Ssttla1, beta_sd_Sdista1, beta_sd_sdung11)

print(betaSD_S104a)

#LEVEL 3. Fit Effects of settlement, grazing and vegetation on GRR burrow density

#Level 3.1: Fit effects of settlement and grazing on GRR via vegetation cover

##Fit with random effect component

GRRhnbmherb04all0 <- glmmTMB(freshholes~settype+distance + dung+vegcvrall +(1|site/transect) ,

data=GRR2021v1F, family=nbinom2,

na.action = na.fail)

r.squaredGLMM(GRRhnbmherb04all0)

#best fit

testDispersion(GRRhnbmherb04all0) #dispersion is fine

testZeroInflation(GRRhnbmherb04all0) #No problem detected

simulationhrp1sA74all0 <- simulateResiduals(fittedModel = GRRhnbmherb04all0, plot = F)

plot(simulationhrp1sA74all0)#fine

plotResiduals(simulationhrp1sA74all0, form = GRR2021v1F$settype) #Within-group deviation detected

plotResiduals(simulationhrp1sA74all0, form = GRR2021v1F$distance)

plotResiduals(simulationhrp1sA74all0, form = GRR2021v1F$dung) #quantile deviation detected

plotResiduals(simulationhrp1sA74all0, form = GRR2021v1F$vegcvrall) #quantile deviations detected

r2(GRRhnbmherb04all0)

summary(GRRhnbmherb04all0)

##Fit without random effect

GRRhnbmherb04all1<- glmmTMB(freshholes~settype+distance + dung+vegcvrall ,

data=GRR2021v1F, family=nbinom2,

na.action = na.fail)

testDispersion(GRRhnbmherb04all1) #dispersion is fine

testZeroInflation(GRRhnbmherb04all1) #No problem detected

simulationhrp1sA74all1 <- simulateResiduals(fittedModel = GRRhnbmherb04all1, plot = F)

plot(simulationhrp1sA74)#quartile devaition detected

plotResiduals(simulationhrp1sA74all1, form = GRR2021v1F$settype) #Within-group devations detected

plotResiduals(simulationhrp1sA74all1, form = GRR2021v1F$distance)

plotResiduals(simulationhrp1sA74all1, form = GRR2021v1F$dung) #quartile deviations detected

plotResiduals(simulationhrp1sA74all1, form = GRR2021v1F$vegcvrall)

r2(GRRhnbmherb04all1)

summary(GRRhnbmherb04all1)

##Fit by including dung and setttype as zi

GRRhnbmherb04all<- glmmTMB(freshholes~settype+distance + dung+vegcvrall ,

zi=~settype+dung,

data=GRR2021v1F, family=nbinom2,

na.action = na.fail)

#best fit

testDispersion(GRRhnbmherb04all) #dispersion is fine

testZeroInflation(GRRhnbmherb04all) #No problem detected

simulationhrp1sA74all <- simulateResiduals(fittedModel = GRRhnbmherb04all, plot = F)

plot(simulationhrp1sA74)#plot(simulationS_norm)

plotResiduals(simulationhrp1sA74all, form = GRR2021v1F$settype)

plotResiduals(simulationhrp1sA74all, form = GRR2021v1F$distance)

plotResiduals(simulationhrp1sA74all, form = GRR2021v1F$dung)

plotResiduals(simulationhrp1sA74all, form = GRR2021v1F$vegcvrall)

r2(GRRhnbmherb04all)

summary(GRRhnbmherb04all)

##Model #GRRhnbmherb04all best fit

#Get standerdized coefficients

R2_GRRvegall <- cor(GRR2021v1F$freshholes, predict(GRRhnbmherb04all, type = "response"))^2

sd.yhat_Grrvegall <- sqrt(var(predict(GRRhnbmherb04all, type = "link"))/R2_GRRvegall) # according to Lefcheck, this approach is also

beta_sd_Grrvegsttla <-fixef(GRRhnbmherb04all)[[1]][2] * sd(as.numeric(GRR2021v1F$settype)[-1L])/sd.yhat_Grrvegall

beta_sd_Grrvegdista <-fixef(GRRhnbmherb04all)[[1]][3] * sd(GRR2021v1F$distance)/sd.yhat_Grrvegall

beta_sd_Grrvegdung <-fixef(GRRhnbmherb04all)[[1]][4] * sd(GRR2021v1F$dung)/sd.yhat_Grrvegall

beta_sd_Grrvegall <-fixef(GRRhnbmherb04all)[[1]][5] * sd(GRR2021v1F$vegcvrall)/sd.yhat_Grrvegall

beta_sdest_Grrvegall <- c(beta_sd_Grrvegsttla, beta_sd_Grrvegdista, beta_sd_Grrvegdung, beta_sd_Grrvegall)

summary(GRRhnbmherb04all)

print(beta_sdest_Grrvegall)

r2(GRRhnbmherb04all)

check_collinearity(GRRhnbmherb04all)

#refit the above using optimizationand with random effect component

GRRhnbmherb04all0optim <- glmmTMB(freshholes~settype+distance + dung+vegcvrall +(1|site/transect) ,

ziformula = ~settype+dung+vegcvrall,

data=GRR2021v1F, family=nbinom2,

na.action = na.fail)

r.squaredGLMM(GRRhnbmherb04all0optim)

#best fit

testDispersion(GRRhnbmherb04all0optim) #dispersion is fine

testZeroInflation(GRRhnbmherb04all0optim) #No problem detected

simulationhrp1sA74all0optim <- simulateResiduals(fittedModel = GRRhnbmherb04all0optim, plot = F)

plot(simulationhrp1sA74all0optim)#fine

plotResiduals(simulationhrp1sA74all0optim, form = GRR2021v1F$settype) #Within-group deviation detected

plotResiduals(simulationhrp1sA74all0optim, form = GRR2021v1F$distance)

plotResiduals(simulationhrp1sA74all0optim, form = GRR2021v1F$dung) #quantile deviation detected

plotResiduals(simulationhrp1sA74all0optim, form = GRR2021v1F$vegcvrall) #quantile deviations detected

r2(GRRhnbmherb04all0optim)

summary(GRRhnbmherb04all0optim)

GRRhnbmherb04all0optim_optim <- update(GRRhnbmherb04all0optim,

control=glmmTMBControl(optimizer=optim,

optArgs=list(method="BFGS")))

summary(GRRhnbmherb04all0optim_optim)

r2(GRRhnbmherb04all0optim_optim)

testDispersion(GRRhnbmherb04all0optim_optim) #dispersion is fine

testZeroInflation(GRRhnbmherb04all0optim_optim) #No problem detected

simulationhrp1sA74all0optim2023 <- simulateResiduals(fittedModel = GRRhnbmherb04all0optim_optim, plot = F)

plot(simulationhrp1sA74all0optim2023)#fine

plotResiduals(simulationhrp1sA74all0optim2023, form = GRR2021v1F$settype) #Within-group deviation detected

plotResiduals(simulationhrp1sA74all0optim2023, form = GRR2021v1F$distance)

plotResiduals(simulationhrp1sA74all0optim2023, form = GRR2021v1F$dung) #quantile deviation detected

plotResiduals(simulationhrp1sA74all0optim2023, form = GRR2021v1F$vegcvrall) #quantile deviations detected

r2(GRRhnbmherb04all0optim)

summary(GRRhnbmherb04all0optim)

###

#Get standerdized coefficients

R2_GRRvegallopt <- cor(GRR2021v1F$freshholes, predict(GRRhnbmherb04all0optim_optim, type = "response"))^2

sd.yhat_Grrvegallopt <- sqrt(var(predict(GRRhnbmherb04all0optim_optim, type = "link"))/R2_GRRvegallopt) # according to Lefcheck, this approach is also

beta_sd_Grrvegsttlaopt <-fixef(GRRhnbmherb04all0optim_optim)[[1]][2] * sd(as.numeric(GRR2021v1F$settype)[-1L])/sd.yhat_Grrvegallopt

beta_sd_Grrvegdistaopt <-fixef(GRRhnbmherb04all0optim_optim)[[1]][3] * sd(GRR2021v1F$distance)/sd.yhat_Grrvegallopt

beta_sd_Grrvegdungopt <-fixef(GRRhnbmherb04all0optim_optim)[[1]][4] * sd(GRR2021v1F$dung)/sd.yhat_Grrvegallopt

beta_sd_Grrvegallopt <-fixef(GRRhnbmherb04all0optim_optim)[[1]][5] * sd(GRR2021v1F$vegcvrall)/sd.yhat_Grrvegallopt

beta_sdest_Grrvegallopt <- c(beta_sd_Grrvegsttlaopt, beta_sd_Grrvegdistaopt, beta_sd_Grrvegdungopt, beta_sd_Grrvegallopt)

summary(GRRhnbmherb04all0optim_optim)

print(beta_sdest_Grrvegallopt)

r2(GRRhnbmherb04all0optim_optim, tolerance = 1e-10)

##Fit with random effect component

#####

#Plots

Grrdung_plot<-plot(ggpredict(GRRhnbmherb04all, terms=c("dung")) %>% plot(rawdata=T, dot.alpha = 1, dot.size=10, color = "black")+#, limit.range=T) +

labs(x="No. dung", y="No. burrow", title="(C)")+

theme_bw() + theme(panel.grid.major = element_blank(),

panel.grid.minor = element_blank(),

panel.border = element_blank(),

axis.line = element_line(colour = "black", size=4),

axis.text = element_text(colour = "black", size=90),

title=element_text(size=90), legend.text = element_text(size=90)) +

scale_x_continuous(breaks=seq(0,250,100)) +

scale_y_continuous(breaks = seq(0,333,300))+ geom_line(size=4))#,face="bold"

Grrdistance_plot<-plot(ggpredict(GRRhnbmherb04all, terms=c("distance")) %>% plot(rawdata=T, dot.alpha = 1, dot.size=10, color = "black")+#, limit.range=T) +

labs(x="Distance (m)", y="No. burrow", title="(A)")+

theme_bw() + theme(panel.grid.major = element_blank(),

panel.grid.minor = element_blank(),

panel.border = element_blank(),

axis.line = element_line(colour = "black", size=4),

axis.text = element_text(colour = "black", size=90),

title=element_text(size=90), legend.text = element_text(size=90)) +

scale_x_continuous(breaks=seq(0,1250,450)) +

scale_y_continuous(breaks=seq(0,333,150))+

geom_line(size=4))#,face="bold"

Grrvegcvrdung_plot<-plot(ggpredict(GRRhnbmherb04all, terms=c("dung")) %>% plot(rawdata=T, dot.alpha = 1, dot.size=10, color = "black")+#, limit.range=T) +

labs(x="No. dung", y=NULL, title="(B)")+

theme_bw() + theme(panel.grid.major = element_blank(),

panel.grid.minor = element_blank(),

panel.border = element_blank(),

axis.line = element_line(colour = "black", size=4),

axis.text = element_text(colour = "black", size=90),

title=element_text(size=90), legend.text = element_text(size=72)) +

scale_x_continuous(breaks=seq(0,250,100)) +

scale_y_continuous(breaks = NULL)+ geom_line(size=4))#,face="bold"

Grrvegcvrvgc_plot<-plot(ggpredict(GRRhnbmherb04all, terms=c("vegcvrall")) %>% plot(rawdata=T, dot.alpha = 1, dot.size=10, color = "black")+#, limit.range=T) +

labs(x="V. cover", y=NULL, title="(C)")+

theme_bw() + theme(panel.grid.major = element_blank(),

panel.grid.minor = element_blank(),

panel.border = element_blank(),

axis.line = element_line(colour = "black", size=4),

axis.text = element_text(colour = "black", size=90),

title=element_text(size=90), legend.text = element_text(size=90)) +

scale_x_continuous(breaks=seq(0,1,0.4)) +

scale_y_continuous(breaks = NULL)+

geom_line(size=4))#,face="bold"

#Level 3.2: effects, via richness, of settlement and grazing on GRR

GRRhnbmS1 = glmmTMB(freshholes~settype+distance + dung+S +(1|site/transect),

data=GRR2021v1F, family=nbinom2)

testDispersion(GRRhnbmS1) #dispersion is fine

testZeroInflation(GRRhnbmS1) #No problem detected

simulationhrp1sA4 <- simulateResiduals(fittedModel = GRRhnbmS1, plot = F)

plot(simulationhrp1sA4)#converged

plotResiduals(simulationhrp1sA4, form = GRR2021v1F$settype) #within group deviation detected

plotResiduals(simulationhrp1sA4, form = GRR2021v1F$distance)

plotResiduals(simulationhrp1sA4, form = GRR2021v1F$dung) #Quantile deviation detected

plotResiduals(simulationhrp1sA4, form = GRR2021v1F$S)

summary(GRRhnbmS1)

r2(GRRhnbmS1)

#Fit without random effect

GRRhnbmS2 = glmmTMB(freshholes~settype+distance + dung+S,

data=GRR2021v1F, family=nbinom2)

testDispersion(GRRhnbmS2) #dispersion is fine

testZeroInflation(GRRhnbmS2) #No problem detected

simulationhrp1saa <- simulateResiduals(fittedModel = GRRhnbmS2, plot = F)

plot(simulationhrp1saa)#converged

plotResiduals(simulationhrp1saa, form = GRR2021v1F$settype) #within group deviation detected

plotResiduals(simulationhrp1saa, form = GRR2021v1F$distance)

plotResiduals(simulationhrp1saa, form = GRR2021v1F$dung) #Quantile deviation detected

plotResiduals(simulationhrp1saa, form = GRR2021v1F$S)

summary(GRRhnbmS2)

r2(GRRhnbmS2)

#Both models had similar AIC and fit

#Thus fit the first model by incorporating settlement type and dung as zi

GRRhnbmS3 = glmmTMB(freshholes~settype+distance + dung+S +(1|site/transect),

zi=~settype +dung,

data=GRR2021v1F, family=nbinom2,

na.action = na.fail) #relatively best fit

testDispersion(GRRhnbmS3) #dispersion is fine

testZeroInflation(GRRhnbmS3) #No problem detected

simulationhrp1sAbb <- simulateResiduals(fittedModel = GRRhnbmS3, plot = F)

plot(simulationhrp1sAbb)#plot(simulationS_norm)

plotResiduals(simulationhrp1sAbb, form = GRR2021v1F$settype)

plotResiduals(simulationhrp1sAbb, form = GRR2021v1F$distance)

plotResiduals(simulationhrp1sAbb, form = GRR2021v1F$dung) #Quantile deviation detected

plotResiduals(simulationhrp1sAbb, form = GRR2021v1F$S)

summary(GRRhnbmS3)

r2(GRRhnbmS3)

#GRRhnbmS3: AIC = 1491.5, R2 = 23.7 vs GRRhnbmS1: AIC = 1525; conditional R2 = 17.7

#Conclusion: use GRRhnbmS3 as best model

GRRhnbmS1

R2_GRRS <- cor(GRR2021v1F$freshholes, predict(GRRhnbmS3, type = "response"))^2

sd.yhat_GRRS <- sqrt(var(predict(GRRhnbmS3, type = "link"))/R2_GRRS) # according to Lefcheck, this approach is also

beta_sd_GRRSsttla <-fixef(GRRhnbmS3)[[1]][2] * sd(as.numeric(GRR2021v1F$settype)[-1L])/sd.yhat_GRRS

beta_sd_GRRSdista <-fixef(GRRhnbmS3)[[1]][3] * sd(GRR2021v1F$distance)/sd.yhat_GRRS

beta_sd_GRRSdung <-fixef(GRRhnbmS3)[[1]][4] * sd(GRR2021v1F$dung)/sd.yhat_GRRS

beta_sd_GRRSS<-fixef(GRRhnbmS3)[[1]][5] * sd(GRR2021v1F$S)/sd.yhat_GRRS

#Get standerdized coefficients

beta_sd_GRRS <- c(beta_sd_GRRSsttla, beta_sd_GRRSdista, beta_sd_GRRSdung, beta_sd_GRRSS)

print(beta_sd_GRRS)

r2(GRRhnbmS3)

summary(GRRhnbmS3)

##Create plots

GrrdisS_plot<-plot(ggpredict(GRRhnbmS3, terms=c("distance")) %>% plot(rawdata=T, dot.alpha = 1, dot.size=10, color = "black")+#, limit.range=T) +

labs(x="Distance (m)", y="No. burrow", title="(A)")+

theme_bw() + theme(panel.grid.major = element_blank(),

panel.grid.minor = element_blank(),

panel.border = element_blank(),

axis.line = element_line(colour = "black", size=4),

axis.text = element_text(colour ="black", size=90),

title=element_text(size=90), legend.text = element_text(size=90))+

scale_x_continuous(breaks=seq(0,1250,500))+

scale_y_continuous(breaks=seq(0,333,200))+

geom_line(size=4))#,face="bold"

GrrdungS_plot<-plot(ggpredict(GRRhnbmS3, terms=c("dung")) %>% plot(rawdata=T, dot.alpha = 1, dot.size=10, color = "black")+#, limit.range=T) +

labs(x="No. dung", y=NULL, title="(B)")+

theme_bw() + theme(panel.grid.major = element_blank(),

panel.grid.minor = element_blank(),

panel.border = element_blank(),

axis.line = element_line(colour = "black", size=4),

axis.text = element_text(colour ="black", size=90),

title=element_text(size=90), legend.text = element_text(size=90))+

scale_x_continuous(breaks = NULL)+

scale_y_continuous(breaks = NULL)+

geom_line(size=4))#,face="bold"

GrrrichS_plot<-plot(ggpredict(GRRhnbmS3, terms=c("S")) %>% plot(rawdata=T, dot.alpha = 1, dot.size=10, color = "black")+#, limit.range=T) +

labs(x="Richness", y=NULL, title="(C)")+

theme_bw() + theme(panel.grid.major = element_blank(),

panel.grid.minor = element_blank(),

panel.border = element_blank(),

axis.line = element_line(colour = "black", size=4),

axis.text = element_text(colour ="black", size=90),

title=element_text(size=90), legend.text = element_text(size=90))+

scale_x_continuous(breaks=seq(0,24,7))+

scale_y_continuous(breaks=NULL)+

geom_line(size=4))#,face="bold"

###################reciprocal effects analysis

#Level 1: effects of settlement type and distance on grazing

# same to above

#LEVEL 2: EXAMINE EFFECTS of settlement, distance and grazing ON GRR burrow density

GRRNB02A = glmmTMB(freshholes~settype+distance + dung +(1|site/transect),

data=GRR2021v1F, family=nbinom2)

testDispersion(GRRNB02A) #dispersion is fine

testZeroInflation(GRRNB02A) #No problem detected

simulationGRRA <- simulateResiduals(fittedModel = GRRNB02A, plot = F)

plot(simulationGRRA)#plot(simulationS_norm)

plotResiduals(simulationGRRA, form = GRR2021v1F$settype)#within-group deviation detected

plotResiduals(simulationGRRA, form = GRR2021v1F$distance)

plotResiduals(simulationGRRA, form = GRR2021v1F$dung)

summary(GRRNB02A)

r2(GRRNB02A)

GRRNB02A0 = glmmTMB(freshholes~settype+distance + dung ,

data=GRR2021v1F, family=gaussian())

testDispersion(GRRNB02A0) #dispersion is fine

testZeroInflation(GRRNB02A0) #No problem detected

simulationGRRA0 <- simulateResiduals(fittedModel = GRRNB02A0, plot = F)

plot(simulationGRRA)#plot(simulationS_norm)

plotResiduals(simulationGRRA0, form = GRR2021v1F$settype)

plotResiduals(simulationGRRA0, form = GRR2021v1F$distance)

plotResiduals(simulationGRRA0, form = GRR2021v1F$dung)

summary(GRRNB02A)

r2(GRRNB02A)

GRRNB02A1 = glmmTMB(freshholes~settype+distance + dung +(1|site/transect),

zi=~dung,

data=GRR2021v1F, family=nbinom2)

testDispersion(GRRNB02A1) #dispersion is fine

testZeroInflation(GRRNB02A1) #No problem detected

simulationGRRA1 <- simulateResiduals(fittedModel = GRRNB02A1, plot = F)

plot(simulationGRRA1)#plot(simulationS_norm)

plotResiduals(simulationGRRA1, form = GRR2021v1F$settype)

plotResiduals(simulationGRRA1, form = GRR2021v1F$distance)

plotResiduals(simulationGRRA1, form = GRR2021v1F$dung)

summary(GRRNB02A1)

GRRNB02A2 = glmmTMB(freshholes~settype+distance + dung,

zi=~settype,

dispformula = ~dung+distance+settype,

data=GRR2021v1F, family=nbinom2())

testDispersion(GRRNB02A2) #dispersion is fine

testZeroInflation(GRRNB02A2) #No problem detected

simulationGRRA2 <- simulateResiduals(fittedModel = GRRNB02A2, plot = F)

plot(simulationGRRA2)#plot(simulationS_norm)

plotResiduals(simulationGRRA2, form = GRR2021v1F$settype)

plotResiduals(simulationGRRA2, form = GRR2021v1F$distance)

plotResiduals(simulationGRRA2, form = GRR2021v1F$dung)

summary(GRRNB02A2)

r2(GRRNB02A2)

#Revise model by including zi and disp formula

GRRNB02A2023 = glmmTMB(freshholes~settype+distance + dung +(1|site/transect),

ziformula = ~settype,

dispformula = ~dung+settype+distance,

data=GRR2021v1F, family=nbinom2) #best fit

testDispersion(GRRNB02A2023) #dispersion is fine

testZeroInflation(GRRNB02A) #No problem detected

simulationGRRA2023 <- simulateResiduals(fittedModel = GRRNB02A2023, plot = F)

plot(simulationGRRA2023)#plot(simulationS_norm)

plotResiduals(simulationGRRA2023, form = GRR2021v1F$settype)#within-group deviation detected

plotResiduals(simulationGRRA2023, form = GRR2021v1F$distance)

plotResiduals(simulationGRRA2023, form = GRR2021v1F$dung)

summary(GRRNB02A2023)

r2(GRRNB02A2023)

#obtain parameter estimates for #GRRNB02A2023

R2_GRR2023 <- cor(GRR2021v1F$freshholes, predict(GRRNB02A2023, type = "response"))^2

sd.yhat_GRR2023 <- sqrt(var(predict(GRRNB02A2023, type = "link"))/R2_GRR2023) # according to Lefcheck, this approach is also

beta_sd_GRRSsttla2023 <-fixef(GRRNB02A2023)[[1]][2] * sd(as.numeric(GRR2021v1F$settype)[-1L])/sd.yhat_GRR2023

beta_sd_GRRSdista2023 <-fixef(GRRNB02A2023)[[1]][3] * sd(GRR2021v1F$distance)/sd.yhat_GRR2023

beta_sd_GRRSdung2023 <-fixef(GRRNB02A2023)[[1]][4] * sd(GRR2021v1F$dung)/sd.yhat_GRR2023

#Get standerdized coefficients

beta_sd_GRR2023 <- c(beta_sd_GRRSsttla2023, beta_sd_GRRSdista2023, beta_sd_GRRSdung2023)

print(beta_sd_GRR2023)

r2(GRRNB02A2023)

summary(GRRNB02A2023)

#fit with site as predictor #poor fit

GRRNB02A2st = glmmTMB(freshholes~settype+distance + dung +site,

zi=~site,

dispformula = ~dung+distance+settype,

data=GRR2021v1F, family=nbinom2())

testDispersion(GRRNB02A2st) #dispersion is fine

testZeroInflation(GRRNB02A2st) #No problem detected

simulationGRRA2st <- simulateResiduals(fittedModel = GRRNB02A2st, plot = F)

plot(simulationGRRA2st)#plot(simulationS_norm)

plotResiduals(simulationGRRA2st, form = GRR2021v1F$settype)

plotResiduals(simulationGRRA2st, form = GRR2021v1F$distance)

plotResiduals(simulationGRRA2st, form = GRR2021v1F$dung)

summary(GRRNB02A2st)

r2(GRRNB02A2st)

##################

#fit with site alone as random predictor #poor fit

GRRNB02A2ste = glmmTMB(freshholes~settype+distance + dung +(1|site),

zi=~settype,

data=GRR2021v1F, family=nbinom2())

testDispersion(GRRNB02A2ste) #dispersion is fine

testZeroInflation(GRRNB02A2ste) #No problem detected

simulationGRRA2ste <- simulateResiduals(fittedModel = GRRNB02A2ste, plot = F)

plot(simulationGRRA2ste)#plot(simulationS_norm)

plotResiduals(simulationGRRA2ste, form = GRR2021v1F$settype)

plotResiduals(simulationGRRA2ste, form = GRR2021v1F$distance)

plotResiduals(simulationGRRA2ste, form = GRR2021v1F$dung)

summary(GRRNB02A2ste)

r2(GRRNB02A2ste)

##################

GRRNB02A3 = glmmTMB(freshholes~settype+distance + dung,

dispformula = ~settype,

data=GRR2021v1F, family=nbinom2())

testDispersion(GRRNB02A3) #dispersion is fine

testZeroInflation(GRRNB02A1) #No problem detected

simulationGRRA3 <- simulateResiduals(fittedModel = GRRNB02A3, plot = F)

plot(simulationGRRA3)#plot(simulationS_norm)

plotResiduals(simulationGRRA3, form = GRR2021v1F$settype)

plotResiduals(simulationGRRA3, form = GRR2021v1F$distance)

plotResiduals(simulationGRRA3, form = GRR2021v1F$dung)

summary(GRRNB02A3)

r2(GRRNB02A3)

GRRNB02A4 = glmmTMB(freshholes~settype+distance + dung+(1|site/transect),

zi=~settype ,

data=GRR2021v1F, family=nbinom2())

testDispersion(GRRNB02A4) #dispersion is fine

testZeroInflation(GRRNB02A4) #No problem detected

simulationGRRA4 <- simulateResiduals(fittedModel = GRRNB02A4, plot = F)

plot(simulationGRRA4)#plot(simulationS_norm)

plotResiduals(simulationGRRA4, form = GRR2021v1F$settype)

plotResiduals(simulationGRRA4, form = GRR2021v1F$distance)

plotResiduals(simulationGRRA4, form = GRR2021v1F$dung)

summary(GRRNB02A4)

r2(GRRNB02A4)

#Conclusion: GRRNB02A2 best fit based on LogLik test

print(R2_dunghnbm1)

R2_GRRNB02A2 <- cor(GRR2021v1F$freshholes, predict(GRRNB02A2, type = "response"))^2

sd.yhat_GRRA2 <- sqrt(var(predict(GRRNB02A2, type = "link"))/R2_GRRNB02A2) # according to Lefcheck, this approach is also

beta_sd_grrA2sttla <-fixef(GRRNB02A2)[[1]][2] * sd(as.numeric(GRR2021v1F$settype)[-1L])/sd.yhat_GRRA2

beta_sd_grrA2dista <-fixef(GRRNB02A2)[[1]][3] * sd(GRR2021v1F$distance)/sd.yhat_GRRA2

beta_sd_grrA2dung <-fixef(GRRNB02A2)[[1]][4] * sd(GRR2021v1F$dung)/sd.yhat_GRRA2

performance_rmse(GRRNB02A2, normalized = TRUE, verbose = TRUE)

check_collinearity(

GRRNB02A2,

component = "all", verbose = TRUE)

beta.sd_grrA2 <- c(beta_sd_grrA2sttla, beta_sd_grrA2dista,beta_sd_grrA2dung)

print(beta.sd_grrA2)

summary(GRRNB02A2)

r2(GRRNB02A2)

###Level 3.1: Effect on vegetation cover

#Fit with random effect

vegcvrall33 <- glmmTMB(vegcvrall~settype+distance + dung +freshholes+(1|site/transect),

data=GRR2021v1F,

na.action = na.fail, family=beta_family())

testDispersion(vegcvrall33) #dispersion is fine

testZeroInflation(vegcvrall33) #No problem detected

simulationherbgrr33 <- simulateResiduals(fittedModel = vegcvrall33, plot = F)

plot(simulationherbgrr2)#plot(simulationS_norm)

plotResiduals(simulationherbgrr33, form = GRR2021v1F$settype)

plotResiduals(simulationherbgrr33, form = GRR2021v1F$distance)#deviation from unifromity detected

plotResiduals(simulationherbgrr33, form = GRR2021v1F$dung) #deviation quantile detected

plotResiduals(simulationherbgrr33, form = GRR2021v1F$freshholes)

summary(vegcvrall33)

r2(vegcvrall33)

#Refit without random effect

vegcvrall33a <- glmmTMB(vegcvrall~settype+distance + dung +freshholes,

data=GRR2021v1F,

na.action = na.fail, family=beta_family())

testDispersion(vegcvrall33a) #dispersion is fine

testZeroInflation(vegcvrall33a) #No problem detected

simulationherbgrr33a <- simulateResiduals(fittedModel = vegcvrall33a, plot = F)

plot(simulationherbgrr33a)#highly skewed

plotResiduals(simulationherbgrr33a, form = GRR2021v1F$settype)

plotResiduals(simulationherbgrr33a, form = GRR2021v1F$distance)#deviation from unifromity detected

plotResiduals(simulationherbgrr33a, form = GRR2021v1F$dung) #deviation quantile detected

plotResiduals(simulationherbgrr33a, form = GRR2021v1F$freshholes)

summary(vegcvrall33a)

r2(vegcvrall33a)

#Get parameter estimates for the model with random component

check_collinearity(vegcvrall33,

component = "all", verbose = TRUE)

###############

R2_vegall <- cor(GRR2021v1F$vegcvrall, predict(vegcvrall33, type = "response"))^2

sd.yhat_vegall3 <- sqrt(var(predict(vegcvrall33, type = "link"))/R2_vegall) # according to Lefcheck, this approach is also

beta_sd_vegGrrsttla <-fixef(vegcvrall33)[[1]][2] * sd(as.numeric(GRR2021v1F$settype)[-1L])/sd.yhat_vegall3

beta_sd_vegGrrdista <-fixef(vegcvrall33)[[1]][3] * sd(GRR2021v1F$distance)/sd.yhat_vegall3

beta_sd_vegGrrdung <-fixef(vegcvrall33)[[1]][4] * sd(GRR2021v1F$dung)/sd.yhat_vegall3

beta_sd_vegGrrgrr <-fixef(vegcvrall33)[[1]][5] * sd(GRR2021v1F$freshholes)/sd.yhat_vegall3

beta.sd_estveg <- c(beta_sd_vegGrrsttla, beta_sd_vegGrrdista,beta_sd_vegGrrdung,beta_sd_vegGrrgrr)

print(beta.sd_estveg)

summary(vegcvrall33)

r2(vegcvrall33)

vegcvrsettl_plot<-plot(ggpredict(vegcvrall33, terms=c("settype")) %>% plot(rawdata=T, dot.alpha = 1, dot.zise=10, color = "black")+#, limit.range=T) +

labs(x="Settlement type", y="Vegetation cover", title="(A)")+

theme_bw() + theme(panel.grid.major = element_blank(),

panel.grid.minor = element_blank(),

panel.border = element_blank(),

axis.line = element_line(colour = "black", size=4),

axis.text = element_text(size=90),

title=element_text(size=90), legend.text = element_text(size=90)))

vegcvrdist_plot<-plot(ggpredict(vegcvrall33, terms=c("distance")) %>% plot(rawdata=T, dot.alpha = 1, dot.size=10, color = "black")+#, limit.range=T) +

labs(x="Distance (m)", y="V. cover", title="(A)")+

theme_bw() + theme(panel.grid.major = element_blank(),

panel.grid.minor = element_blank(),

panel.border = element_blank(),

axis.line = element_line(colour = "black", size=4),

axis.text = element_text(colour = "black", size=90),

title=element_text(size=90), legend.text = element_text(size=90)) +

scale_y_continuous(breaks=seq(0,1,0.4))+

scale_x_continuous(breaks = seq(0,1250,450)) +

geom_line(size=4))#,face="bold"

vegcvrdungs_plot<-plot(ggpredict(vegcvrall33, terms=c("dung")) %>% plot(rawdata=T, dot.alpha = 1, dot.size=10, color = "black")+#, limit.range=T) +

labs(x="No. dung", y=NULL, title="(B)")+

theme_bw() + theme(panel.grid.major = element_blank(),

panel.grid.minor = element_blank(),

panel.border = element_blank(),

axis.line = element_line(colour = "black", size=4),

axis.text = element_text(colour = "black", size=90),

title=element_text(size=90), legend.text = element_text(size=90)) +

scale_y_continuous(breaks=NULL)+

scale_x_continuous(breaks = seq(0,250,100)) +

geom_line(size=4))#,face="bold"

vegcvrgrr_plot<-plot(ggpredict(vegcvrall33, terms=c("freshholes")) %>% plot(rawdata=T, dot.alpha = 1, dot.size =10, color = "black")+#, limit.range=T) +

labs(x="No. burrow", y=NULL, title="(C)")+

theme_bw() + theme(panel.grid.major = element_blank(),

panel.grid.minor = element_blank(),

panel.border = element_blank(),

axis.line = element_line(colour = "black", size=4),

axis.text = element_text(colour = "black", size=90),

title=element_text(size=90), legend.text = element_text(size=90)) +

scale_y_continuous(breaks= NULL) +

scale_x_continuous(breaks = seq(0,330,100))+

geom_line(size=4))#,face="bold"

#Level 3.2: Richness as response

#Fit with random effect

S3a01 = glmmTMB(S~settype+distance + dung +freshholes +(1|site/transect) ,

data=GRR2021v1F, family=nbinom2) #did not converge

#Fit without random effect

S3a02 = glmmTMB(S~settype+distance + dung +freshholes ,

data=GRR2021v1F, family=nbinom2)

testDispersion(S3a02) #dispersion is fine

testZeroInflation(S3a02) #No problem detected

simulationhrp1s3a02 <- simulateResiduals(fittedModel = S3a02, plot = F)

plot(simulationhrp1s3a02)#fine

plotResiduals(simulationhrp1s3a02, form = GRR2021v1F$settype)

plotResiduals(simulationhrp1s3a02, form = GRR2021v1F$distance) #deviation detected

plotResiduals(simulationhrp1s3a02, form = GRR2021v1F$dung) #deviation detected

plotResiduals(simulationhrp1s3a02, form = GRR2021v1F$freshholes)

summary(S3a02)

r2(S3a02)

####Fit using gaussian distribution

S3a1 = glmmTMB(S~settype+distance + dung +freshholes ,

zi=~1, data=GRR2021v1F, family=gaussian(), na.action = na.fail)

r.squaredGLMM(S3a1)

testDispersion(S3a1) #dispersion is fine

testZeroInflation(S3a1) #No problem detected

simulationhrp1s3a1 <- simulateResiduals(fittedModel = S3a1, plot = F)

plot(simulationhrp1s3a1)#Better

plotResiduals(simulationhrp1s3a1, form = GRR2021v1F$settype)

plotResiduals(simulationhrp1s3a1, form = GRR2021v1F$distance)

plotResiduals(simulationhrp1s3a1, form = GRR2021v1F$dung)

plotResiduals(simulationhrp1s3a1, form = GRR2021v1F$freshholes)

model_performance(S3a1, metrics = "all")

summary(S3a1)

r2(S3a1)

check_collinearity(S3a1)

#Conclusion: model S3a1 best fit

########Fit with site #different and better fit

#Get parameter estimates

check_collinearity( S3a1, component = "all", verbose = TRUE)

R2_S3a1 <- cor(GRR2021v1F$dung, predict(S3a1, type = "response"))^2

sd.yhat_s3a1 <- sqrt(var(predict(S3a1, type = "link"))/R2_S3a1) # according to Lefcheck, this approach is also

beta_sd_S3a1sett <-fixef(S3a1)[[1]][2] * sd(as.numeric(GRR2021v1F$settype)[-1L])/sd.yhat_s3a1

beta_sd_s3a1dista <-fixef(S3a1)[[1]][3] * sd(GRR2021v1F$distance)/sd.yhat_s3a1

beta_sd_s3a1dung <-fixef(S3a1)[[1]][3] * sd(GRR2021v1F$dung)/sd.yhat_s3a1

beta_sd_s3a1fresh <-fixef(S3a1)[[1]][3] * sd(GRR2021v1F$freshholes)/sd.yhat_s3a1

beta_sd_Richness <- c(beta_sd_S3a1sett, beta_sd_s3a1dista,beta_sd_s3a1dung, beta_sd_s3a1fresh)

r2(S3a1)

summary(S3a1)

print(beta_sd_Richness)

#Fit using random effect and optimization

S3a1RE = glmmTMB(S~settype+distance + dung +freshholes +(1|site/transect),

dispformula = ~dung,

data=GRR2021v1F, family=nbinom2(), na.action = na.fail) #did not converge

#Fit with optimization, or udate model

S3a1RE_optim <- update(S3a1RE,

control=glmmTMBControl(optimizer=optim,

optArgs=list(method="BFGS")))

testDispersion(S3a1RE_optim) #dispersion is fine

testZeroInflation(S3a1RE_optim) #No problem detected

simulationhrp1s3aopt1 <- simulateResiduals(fittedModel = S3a1RE_optim, plot = F)

plot(simulationhrp1s3aopt1)#Better

plotResiduals(simulationhrp1s3aopt1, form = GRR2021v1F$settype)

plotResiduals(simulationhrp1s3aopt1, form = GRR2021v1F$distance)

plotResiduals(simulationhrp1s3aopt1, form = GRR2021v1F$dung)

plotResiduals(simulationhrp1s3aopt1, form = GRR2021v1F$freshholes)

summary(S3a1RE_optim)

r2(S3a1RE_optim, tolerance = 1e-10)

##Conclusion: model S3a1RE_optim best fit

#Get parameter estimates

R2_S3a1opt <- cor(GRR2021v1F$S, predict(S3a1RE_optim, type = "response"))^2

sd.yhat_s3a1opt <- sqrt(var(predict(S3a1RE_optim, type = "link"))/R2_S3a1opt) # according to Lefcheck, this approach is also

beta_sd_S3a1settopt <-fixef(S3a1RE_optim)[[1]][2] * sd(as.numeric(GRR2021v1F$settype)[-1L])/sd.yhat_s3a1opt

beta_sd_s3a1distaopt <-fixef(S3a1RE_optim)[[1]][3] * sd(GRR2021v1F$distance)/sd.yhat_s3a1opt

beta_sd_s3a1dungopt <-fixef(S3a1RE_optim)[[1]][4] * sd(GRR2021v1F$dung)/sd.yhat_s3a1opt

beta_sd_s3a1freshopt <-fixef(S3a1RE_optim)[[1]][5] * sd(GRR2021v1F$freshholes)/sd.yhat_s3a1opt

beta_sd_Richnessopt <- c(beta_sd_S3a1settopt, beta_sd_s3a1distaopt,beta_sd_s3a1dungopt, beta_sd_s3a1freshopt)

r2(S3a1RE_optim, tolerance = 1e-10)

summary(S3a1RE_optim)

print(beta_sd_Richnessopt)

#####

#Create plots

Sgrrdist_plot<-plot(ggpredict(S3a1, terms=c("distance")) %>% plot(rawdata=T, dot.alpha = 1, dot.size=10, color = "black")+#, limit.range=T) +

labs(x="Distance (m)", y="Richnness", title="(A)")+

theme_bw() + theme(panel.grid.major = element_blank(),

panel.grid.minor = element_blank(),

panel.border = element_blank(),

axis.line = element_line(colour = "black", size=4),

axis.text = element_text(colour = "black", size=90),

title=element_text(size=90), legend.text = element_text(size=90)) +

scale_y_continuous(breaks = seq(2,25,10))+

scale_x_continuous(breaks = seq(0,1250,450)) +

geom_line(size=4))#,face="bold"

Sgrrdung_plot<-plot(ggpredict(S3a1, terms=c("dung")) %>% plot(rawdata=T, dot.alpha = 1, dot.size=10, color = "grey20")+#, limit.range=T) +

labs(x="No. dung", y=NULL, title="(B)")+

theme_bw() + theme(panel.grid.major = element_blank(),

panel.grid.minor = element_blank(),

panel.border = element_blank(),

axis.line = element_line(colour = "black", size=4),

axis.text = element_text(colour = "black", size=90),

title=element_text(size=90), legend.text = element_text(size=90)) +

scale_y_continuous(breaks = NULL) +

scale_x_continuous(breaks = seq(0,250,100)) +

geom_line(size=4))#,face="bold"

Sgrr_plot<-plot(ggpredict(S3a1, terms=c("freshholes")) %>% plot(rawdata=T, dot.alpha = 1, dot.size=10, color = "black")+#, limit.range=T) +

labs(x="No. burrow", y=NULL, title="(C)")+

theme_bw() + theme(panel.grid.major = element_blank(),

panel.grid.minor = element_blank(),

panel.border = element_blank(),

axis.line = element_line(colour = "black", size=4),

axis.text = element_text(colour = "black", size=90),

title=element_text(size=90), legend.text = element_text(size=90)) +

scale_y_continuous(breaks = NULL) +

geom_line(size=4))#,face="bold"

################################### the end #################
